# Terbium and Vanadium Metal Nanoparticles Reactive Starting Materials for Liquid‐Phase Syntheses

**DOI:** 10.1002/smll.202503498

**Published:** 2025-06-09

**Authors:** Andreas Reiß, Anja Appenzeller, Jule J. Baur, Jonas O. Wenzel, Radian Popescu, Kathrin Beuthert, Stefanie Dehnen, Yolita M. Eggeler, Frank Breher, Wim Klopper, Claus Feldmann

**Affiliations:** ^1^ Institute of Inorganic Chemistry Karlsruhe Institute of Technology (KIT) Engesserstrasse 15 D‐76131 Karlsruhe Germany; ^2^ Institute of Physical Chemistry Karlsruhe Institute of Technology (KIT) Fritz‐Haber‐Weg 2 D‐76131 Karlsruhe Germany; ^3^ Laboratory for Electron Microscopy Karlsruhe Institute of Technology (KIT) Engesserstrasse 7 D‐76131 Karlsruhe Germany; ^4^ Institute of Nanotechnology Karlsruhe Institute of Technology (KIT) Kaiserstrasse 12 76131 Karlsruhe Germany

**Keywords:** carbonyl, crystal structure analysis, cyclopentadienyl, low‐valence compounds, metal‐metal bonding, terbium nanoparticles, vanadium nanoparticles

## Abstract

Lanthanide metals and early transition metals – although in principle highly reactive – only show a limited reactivity due to small surface, low solubility, and/or passivation. To this regard, small‐sized metal nanoparticles can give the opportunity for reactions near room temperature in the liquid phase. With terbium‐metal nanoparticles (2.8 ± 0.4 nm) and vanadium‐metal nanoparticles (1.2±0.2 nm), representative lanthanide and early‐transition metals are presented with different reactivity. Both are prepared by reduction of simple precursors (TbCl_3_, VCl_3_) in THF. The Tb(0)/V(0) nanoparticles are highly reactive and used as starting materials in the liquid phase (THF, toluene, *n*‐dodecane, ionic liquid) to perform reactions with cyclopentadienyl precursors [Cp_2_ *M*Cl_2_] and carbonyl precursors [*M*(CO)_6_] (*M* = Mo, W). As a result, the novel compounds [BMIm][Cp_2_Mo(GaCl_3_)_2_] **1**), [BMIm][Cp_2_W(GaCl_3_)_2_] **2**), [Cp_2_Mo{GaCl_2_(THF)}_2_] **3**), [BMIm][Cp_2_MoGa_2_Cl_5_] **4**), [VO(H_2_Cyclal)Mo(CO)_4_] **5**) and [VO(H_2_Cyclal)W(CO)_4_] **6**) are obtained, containing metal‐metal bonding (Mo–Ga, W–Ga) and/or low‐valent metals (Mo(0/I), W(0/I), Ga(III)). Profound characterization of structure and bonding is performed (including TEM, XRD, FT‐IR, DFT, MS, and ESR). Tb(0)/V(0) nanoparticles, in general, offer high potential for reactions/compounds different from the bulk lanthanide/transition metals and, specifically, for obtaining metal‐metal bonding and low‐valent metal compounds via a novel redox approach.

## Graphical abstract

Reactive vanadium‐ and terbium‐metal nanoparticles are prepared in THF. Due to their size and reactivity, they offer unique options for liquid‐phase syntheses, e.g., of low‐valence compounds or metal‐metal bonding.

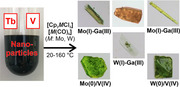

## 1 Introduction

The early transition metals and the lanthanide metals are highly reactive, in principle.^[^ ^1^ ^]^ The expected reactivity is qualitatively indicated by the position of these metals in the voltage series with standard potentials below –1.5 V^[^ ^2^ ^]^ and by the high lattice energy of the respective metal oxides (up to –1,800 kJ mol^−1^).^[^ ^3^ ^]^ In contrast to the expectation, however, the bulk transition/lanthanide metals usually show only low reactivity. This can be attributed to their low solubility, low surface area, and/or passivated surfaces (e.g., by metal hydroxide, oxide, and/or carbonate).^[^ ^4^ ^]^

As an alternative to bulk metals, nanosized metals can be an option to increase chemical reactivity. Metal nanoparticles have a high surface area and a great number of insufficiently coordinated surface atoms. Passivation is usually not possible as the particle diameter is below the thickness of the passivation layer (≈10 nm).^[^ ^5^ ^]^ The knowledge of small‐sized metal nanoparticles, however, is the lower the more negative the electrochemical potential of the respective metal. Taking terbium nanoparticles, Tb(0), and vanadium nanoparticles, V(0), as examples of lanthanide and early transition metals with different reactivity, current knowledge is mainly limited to gas‐phase methods for V(0) nanoparticles,^[^ ^6^ ^]^ while a synthesis of Tb(0) nanoparticles is lacking completely.

Here, we show the liquid‐phase synthesis of Tb(0) and V(0) nanoparticles with diameters of 1‐5 nm. They show high reactivity, as indicated by instantaneous combustion in air or explosion when in contact to water. As indicated by the electrochemical potential of the bulk metals (*E^0^* (V/V^3+^) = –0.87 V; *E^0^* (Tb/Tb^3+^) = –2.3 V),^[^ ^7^ ^]^ the Tb(0) nanoparticles are more reactive than V(0) nanoparticles. Qualitatively, Tb(0) nanoparticles show a reactivity in air or with water similar to that of the heavy alkali metals rubidium and cesium, whereas V(0) nanoparticles behave like bulk sodium. Here, we specifically aim at using Tb(0)/V(0) nanoparticles as starting materials in the liquid phase. To probe the reactivity of base‐metal nanoparticles, we already performed reactions with small molecules (e.g., O_2_, NH_3_, S_8_, ROH, HCp)^[^ ^8^ ^]^ as well as with sterically demanding O–H‐acidic alcohols and N–H‐acidic amines.^[^ ^9^ ^]^ For the first time, we here examine the reactivity of base‐metal nanoparticles in regard of the formation of compounds with metal‐metal bonding and/or low‐valent oxidation states. To this concern, the more reactive Tb(0) and the less reactive V(0) nanoparticles were reacted with cyclopentadienyl precursors [Cp_2_ *M*Cl_2_] and carbonyl precursors [*M*(CO)_6_] (*M* = Mo, W), resulting in the new compounds [BMIm][Cp_2_Mo(GaCl_3_)_2_] (**1**), [BMIm][Cp_2_W(GaCl_3_)_2_] (**2**), [Cp_2_Mo{GaCl_2_(THF)}_2_] (**3**), [BMIm][Cp_2_MoGa_2_Cl_5_] (**4**), [VO(H_2_Cyclal)Mo(CO)_4_] (**5**) and [VO(H_2_Cyclal)W(CO)_4_] (**6**).

## 2 Results and Discussion

### 2.1 Synthesis of Tb(0) and V(0) Nanoparticles

**Figure 1:**
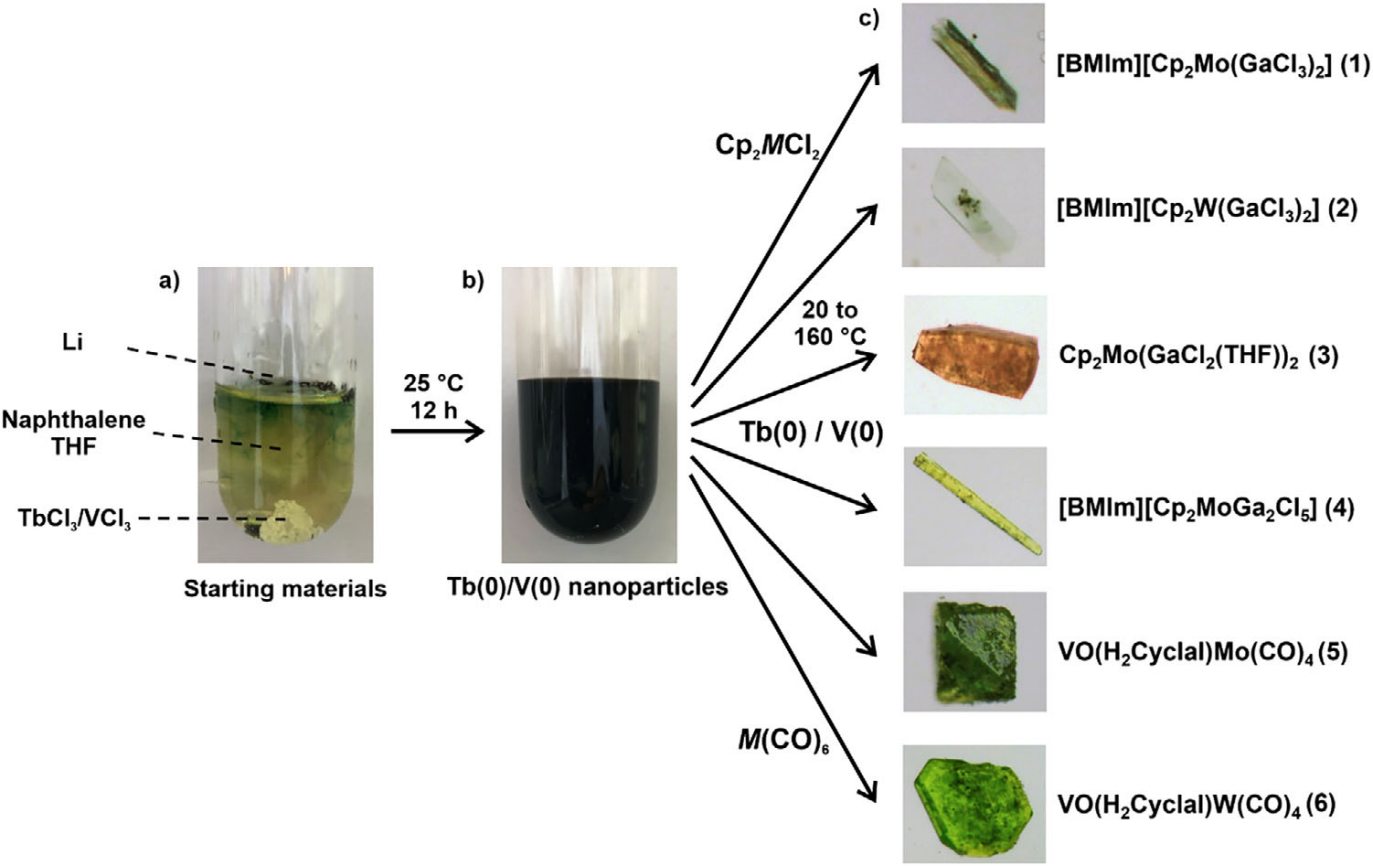
Scheme illustrating the synthesis of Tb(0) and V(0) nanoparticles: a) one‐pot approach with the starting materials, b) as‐prepared Tb(0)/V(0) nanoparticles in THF, c) Tb(0)/V(0) nanoparticles as starting materials to obtain the novel compounds **1‐6**.

The synthesis of zerovalent terbium‐ and vanadium‐metal nanoparticles (Tb(0) and V(0) nanoparticles) was performed following our previously published approach to reduce metal halides with alkali‐metal napthalenides.^[^ ^8^, ^10^ ^]^ Thus, TbCl_3_ or VCl_3_, lithium, and naphthalene were added to THF and stirred for 12 h at room temperature (**Figure** **1**a). This one‐pot approach was selected due to the low solubility of TbCl_3_ and VCl_3_ in THF.^[^ ^11^ ^]^ Upon intense stirring for 12 h, first of all, lithium reacts with naphthalene to form lithium naphthalenide ([LiNaph]).^[^ ^12^ ^]^ This can be followed by the naked eye and the formation of a deep‐green solution. Thereafter, [LiNaph] as a powerful reducing agent reduces all dissolved TbCl_3_ or VCl_3_ instantaneously to elemental terbium and vanadium. Despite of gas‐phase methods,^[^ ^6^ ^]^ V(0) nanoparticles were barely reported and yet required the presence of strong‐binding N‐containing ligands (i.e., pyridine, 4‐octylphenyldiazonium).^[^ ^13^ ^]^ Tb(0) nanoparticles are realized in the liquid phase for the first time.

At first sight, the conditions of the aforementioned one‐pot approach seem disadvantageous for controlling the nucleation of small‐sized Tb(0)/V(0). Several conditions are nevertheless very advantageous: *i*) the dissolution of the metal chlorides is very slow, whereas the reduction of Tb^3+^/V^3+^ by [LiNaph] is very fast; *ii*) the elemental metals are highly insoluble in THF, which causes a high supersaturation. These conditions promote the formation of high‐quality Tb(0)/V(0) nanoparticles (Figure 1b). The as‐prepared metal nanoparticles were purified by centrifugation and repeated redispersion/centrifugation in/from THF to remove the remaining starting materials, naphthalene, and LiCl. Thereafter, the Tb(0)/V(0) nanoparticles were either dried in vacuum at room temperature to obtain powder samples, or they were redispersed in THF or toluene to obtain suspensions that were colloidally stable over some hours. Specific attention must be paid to the synthesis and handling of base‐metal nanoparticles as they show instantaneous combustion in air and even explosion when in contact to water or other oxidizing agents (see Video SV1, Supporting Information). This holds especially for the Tb(0) nanoparticles that exhibit a reactivity similar to that of the heavy alkali metals rubidium or cesium.

**Figure 2:**
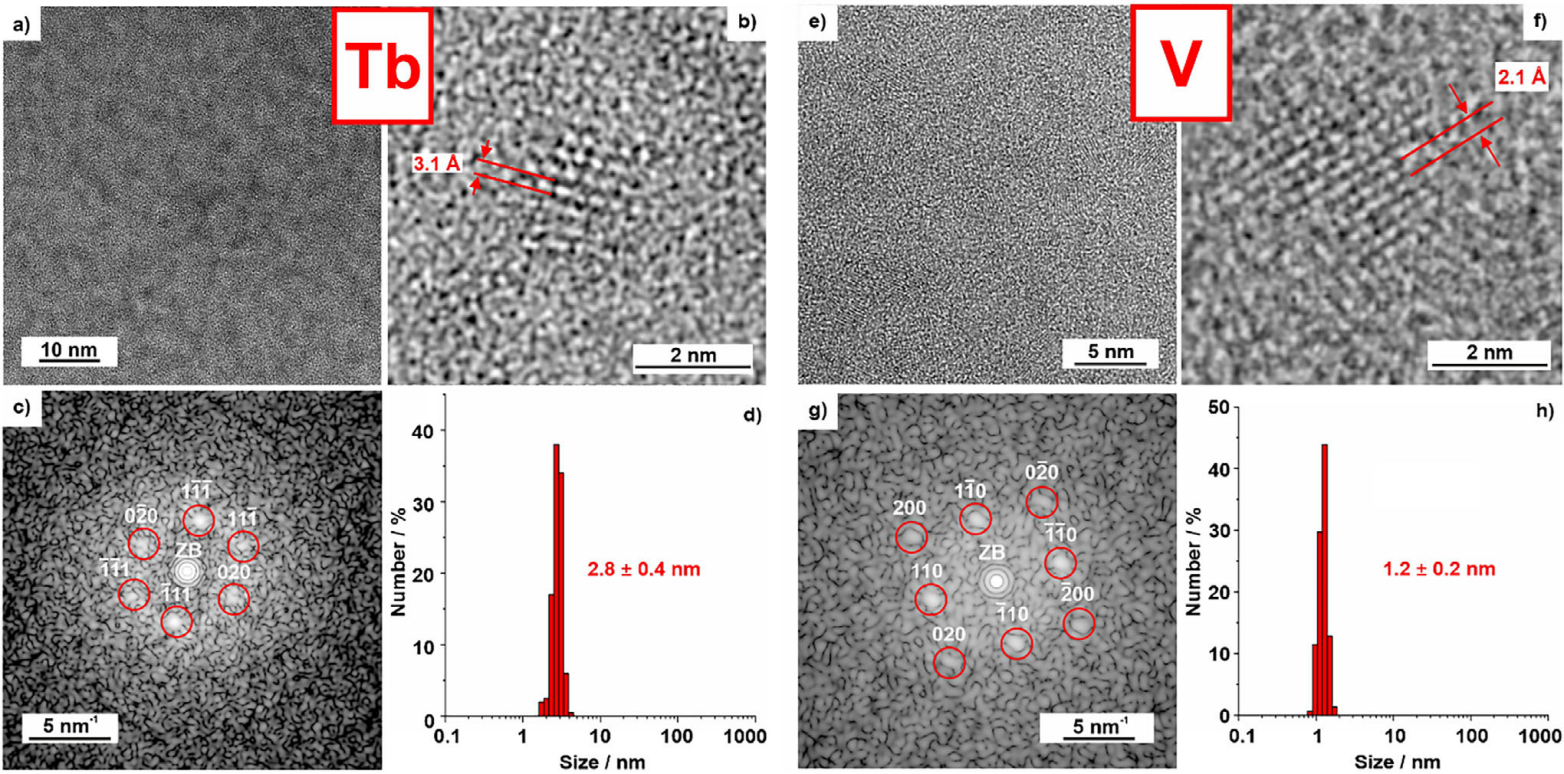
Size and size distribution of the as‐prepared Tb(0) and V(0) nanoparticles: a,e) TEM overview image, b,f) HRTEM image of monocrystalline Tb(0)/V(0) nanoparticle with lattice fringes, c,g) FT analysis of the particle in (b,f) with calculated diffraction patterns and Miller indices of face‐centred cubic bulk terbium in the [101]‐zone axis (c) and face‐centred cubic bulk vanadium in the [001]‐zone axis (g), respectively (zero‐order beam (ZB) indicated by white circle), d,h) size distribution (based on statistical evaluation of > 200 nanoparticles on TEM images).

The size, shape, size distribution, and crystallinity of the as‐prepared Tb(0) and V(0) nanoparticles were examined by transmission electron microscopy (TEM). Accordingly, spherical nanoparticles with uniform shape, a size range of 1‐5 nm, and a low degree of agglomeration were obtained (**Figure** **2**a,e). Statistical evaluation of >200 different nanoparticles on TEM images resulted in a mean particle diameter of 2.8 ± 0.4 nm (Tb(0), Figure 2d) and 1.2±0.2 nm (V(0), Figure 2h), which was also confirmed by dynamic light scattering (Figure S1, Supporting Information). High‐resolution (HR)TEM images confirm the presence and monocrystallinity of the Tb(0) and V(0) nanoparticles, which show lattice fringes extending through the whole particle (Figure 2b,f). In the case of terbium, the observed lattice‐plane distance of 3.1± 0.1 Å is in good agreement with face‐centered cubic bulk terbium *(d*_‐111_ = 3.0 Å).^[^ ^14^ ^]^ For vanadium, the observed lattice‐plane distance of 2.1±0.1 Å is in good agreement with cubic bulk vanadium (*d*_110_ with 2.1 Å).^[^ ^15^ ^]^ The presence of zerovalent terbium and vanadium was further confirmed by the 2D Fourier transformation (FT) analysis of the single, monocrystalline Tb(0) and V(0) nanoparticles on HRTEM images (Figure 2c,g), which for both is in good agreement with the calculated diffraction pattern of face‐centred cubic bulk terbium (space group *Fm*‐3*m*, in the [101]‐zone axis; Figure 2c) and body‐centred cubic bulk vanadium (space group *Im*‐3*m* in the [001]‐zone axis; Figure 2g).

In regard of the reactivity and reactions of the as‐prepared Tb(0) and V(0) nanoparticles, their surface functionalization is relevant. For this purpose, Fourier‐transform infrared (FT‐IR) spectroscopy and elemental analysis (EA) were performed with powder samples. FT‐IR spectra show weak vibrations related to THF (*ν*(C–H): 3000‐2800 cm^−1^, *ν*(C–O): 1050‐800 cm^−1^), which, as expected, is adsorbed on the surface of the metal nanoparticles (Figure S2, Supporting Information). Furthermore, a series of sharp vibrations occurs at 1600‐800 cm^−1^ that can be related to naphthalene, which is also adsorbed on the surface of the Tb(0)/V(0) nanoparticles in addition to THF. This situation, as indicated by FT‐IR spectra, is also confirmed by EA. Here, C/H/N contents of 27.9 wt‐% C, 3.3 wt‐% H (Tb(0)) and 53.4 wt‐% C, 4.1 wt‐% H (V(0)) were obtained, respectively. The adsorption of THF and naphthalene on the particle surface is also confirmed by a C : H ratio of 8 (Tb(0)) and 13 (V(0)), which is higher than the expected C : H ratio for THF (6) but lower than expected for naphthalene (15).

### 2.2 Reactions with Cyclopentadienyl Precursors [Cp_2_MCl_2_] (M: Mo, W)

While the high reactivity of the as‐prepared Tb(0) and V(0) nanoparticles requires special attention during handling and characterization (see Video SV1, Supporting Information), this reactivity also offers the opportunity to use the metal nanoparticles as starting materials in reactions at moderate temperatures (≤100 °C) in the liquid phase that would be hardly possible with bulk terbium or bulk vanadium. Aiming at reactions with cyclopentadienyl precursors [Cp_2_ *M*Cl_2_] and carbonyl precursors [*M*(CO)_6_] (*M* = Mo, W) (Figures S3–S21, Supporting Information), we first examined syntheses of the more reactive Tb(0) nanoparticles with [Cp_2_ *M*Cl_2_] at 50 °C in [BMIm][GaCl_4_] as an ionic liquid. Ionic liquids were used as solvents due to their high chemical and thermal stability.^[^ ^16^ ^]^ This results in the formation of colorless, moisture‐ and air‐sensitive crystals of [BMIm][Cp_2_Mo(GaCl_3_)_2_] (**1**) and [BMIm][Cp_2_W(GaCl_3_)_2_] (**2**) with a yield of ≈40 % related to the initial amount of Tb(0) nanoparticles, according to the following reaction:

(1) $$\begin{array}{ccc} \mathrm{Tb}(0) & + & {\mathrm{Cp}}_{3}{\mathrm{MCl}}_{2}+2\;{\mathrm{GaCl}}_{3}+\left[\mathrm{BMIm}\right]\mathrm{Cl}\;\\ & & \to \;\left[\mathrm{BMIm}\right]\left[{\mathrm{Cp}}_{2}M{\left({\mathrm{GaCl}}_{3}\right)}_{2}\right]+{\mathrm{TbCl}}_{3}\;\left(M:\;\mathrm{Mo},\;W\right) \end{array}$$

Obviously, the Tb(0) nanoparticles “only” serve as reducing agent with terbium not being present in the product. However, it should be noticed that no comparable reaction or product was obtained with bulk sodium as a reducing agent. Thus, the reactivity and reactions of the Tb(0) nanoparticles as a powerful reducing agent in suspension suggest a different behavior possibly related to a surface‐mediated reaction with small particles in the liquid phase.

**Figure 3:**
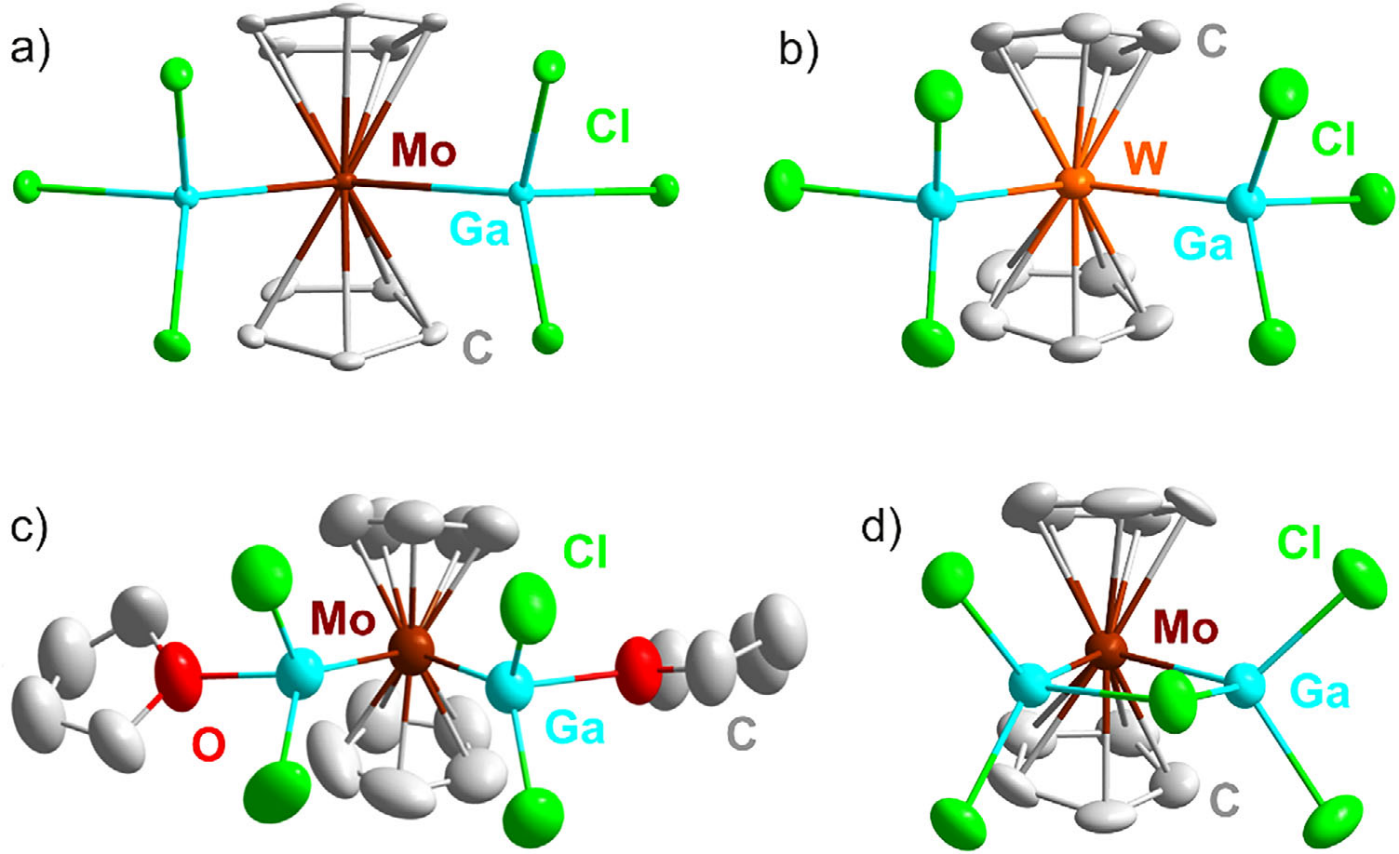
Structures of: a) [Cp_2_Mo(GaCl_3_)_2_]^–^ anion in **1**, b) [Cp_2_W(GaCl_3_)_2_]^–^ anion in **2**, c) [Cp_2_Mo{GaCl_2_(THF)}_2_] (**3**), d) [Cp_2_MoGa_2_Cl_5_]^–^ anion in **4** (H atoms not shown for clarity; data collection for **1** on StadiVari Diffractometer and for **2‐4** on IPDS II diffractometer; *see SI*).

According to single‐crystal structure analysis, **1** and **2** crystallize in the monoclinic space group *P*2_1_ */n* (Table S1 and Figure S3, Supporting Information). They consist of [Cp_2_ *M*(GaCl_3_)_2_]^–^ anions and [BMIm]^+^ cations (**Figure** **3**a,b). The [Cp_2_ *M*(GaCl_3_)_2_]^–^ anions contain a central Cp_2_ *M* unit (*M*: Mo, W) coordinated by two GaCl_3_ groups with unsupported *M*–Ga metal‐metal binding. The complexes, thus, belong to the class of metal‐only Lewis pairs^[^ ^17^ ^]^ with GaCl_3_ serving as *Z*‐type ligand.^[^ ^18^ ^]^ Mo/W exhibit a distorted tetrahedral coordination by two Cp ligands and two Ga atoms. Likewise, the Ga atoms are tetrahedrally coordinated by a Mo/W atom and three Cl atoms. The Mo/W–Ga distances are 261.1(1) and 261.5(1) pm (**1**) as well as 262.9(1) and 263.0(1) pm (**2**) (**Table** **1**). The Mo–C distances in **1** range from 228.9(2)‐232.2(2) pm, the Ga–Cl distances from 222.1(1)‐225.4(1) pm. Similarly, the W–C distances in **2** range from 229.0(6)‐233.7(6) pm, the Ga–Cl distances from 223.2(2)‐226.4(2) pm. Angles of Ga–Mo–Ga (109.3(1) °), Cp–Mo–Cp (141.7(1) °), Cl–Ga–Cl (101.5(1)‐104.3(1) °) and Mo–Ga–Cl (113.6(1)‐117.6(1) °) point to the distorted tetrahedral coordination with Cp–Mo–Cp as the widest angle due to the size of the Cp ligands. A similar situation is found for **2** with Ga–W–Ga (111.1(1) °), Cp–W–Cp (140.9(1) °), Cl–Ga–Cl (101.4(1)‐103.9(1) °) and W–Ga–Cl (115.0(1)‐116.8(1) °).

**Table 1:** Mo–Ga and W–Ga distances (in pm) in **1**‐**4** in comparison to the literature.

Compound	Mo–Ga /pm	W–Ga /pm
[BMIm][Cp_2_Mo(GaCl_3_)_2_] (**1**)	261.1(1)‐261.5(1)	/
[BMIm][Cp_2_W(GaCl_3_)_2_] (**2**)	/	262.9(1)‐263.0(1)
[Cp_2_Mo{GaCl_2_(THF)}_2_] (**3**)	252.9(1)‐253.5(1) pm	/
[BMIm][Cp_2_MoGa_2_Cl_5_] (**4**)	254.1(1)	/
[fac‐(Cp*Ga)_3_M(CO)_3_] [22]	251.9‐252.3	251.9‐252.2
[(OC)_2_(Cp)M{μ^2^‐(η^1^‐GaCp*)}]2[22]	253.7‐260.6	253.6‐260.8
[{(C_5_H_5_)W(CO)_3_}_3_Ga] [24]	/	271.6‐275.8
[Mo(η‐C_5_H_4_Me)(CO)_3_GaI_2_·Et_2_O] [29]	258.2	/

Since the Tb(0) nanoparticles react with the ionic liquid as indicated by a change of color (from colorless to dark brown), a significantly increased viscosity and the limited yield (≈40%) of the title compounds **1** and **2**, [BMIm]Cl and GaCl_3_ were next added in stoichiometric amounts only. Moreover, THF was added as a solvent to guarantee a sufficiently high solubility of TbCl_3_. With such reaction of Tb(0) nanoparticles with Cp_2_MoCl_2_, [BMIm]Cl, and GaCl_3_ in THF at 25 °C, yellow‐brown crystals of [Cp_2_Mo{GaCl_2_(THF)}_2_] (**3**) were obtained in quantitative yield according to the following equation:

(2) $$\begin{array}{ccc} 4\;\mathrm{Tb}(0) & + & 3\;{\mathrm{Cp}}_{2}{\mathrm{MoCl}}_{2}+6\;{\mathrm{GaCl}}_{3}+6\;\mathrm{THF}\;\\ & & \to \;3\;\left[{\mathrm{Cp}}_{2}\mathrm{Mo}{\left\{{\mathrm{GaCl}}_{2}\left(\mathrm{THF}\right)\right\}}_{2}\right]+4\;{\mathrm{TbCl}}_{3} \end{array}$$

**Figure 4:**
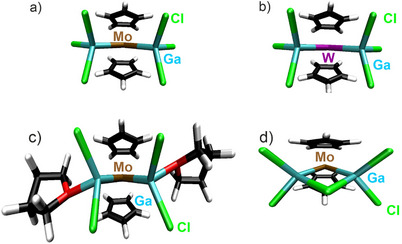
Optimized equilibrium geometries at the B3LYP/dhf‐TZVP‐2c level for a) [Cp_2_Mo(GaCl_3_)_2_]^–^, b) [Cp_2_W(GaCl_3_)_2_]^–^, c) [Cp_2_Mo{GaCl_2_(THF)}_2_], d) [Cp_2_MoGa_2_Cl_5_]^–^.

Although not being part of the product's composition, the addition of [BMIm]Cl is required to obtain crystals of **3**. This can be attributed to the higher solubility of [BMIm][GaCl_4_] as compared to that of GaCl_3_. Terbium still serves as a reducing agent. Furthermore, the more polar solvent THF is present as a ligand coordinating gallium. According to single‐crystal structure analysis, **3** crystallizes in the non‐inversion symmetric space group *P*4_1_ (Table S1 and Figure S4, Supporting Information). The compound consists of non‐charged [Cp_2_Mo{GaCl_2_(THF)}_2_] molecules (**Figure** **4**c). Herein, a central Cp_2_Mo unit is distorted tetrahedrally coordinated by two Ga atoms and two Cp ligands. The Mo–Ga distances are 252.9(1) and 253.5(1) pm. The Mo–C distances range at 227.4(10)‐232.5(9) pm. The Ga atoms are also distorted tetrahedrally coordinated by the Mo atom, two Cl atoms, and one O atom of a THF molecule. One of the THF molecules shows positional disorder, which was addressed by split‐atom positions with an occupancy of 50% for each atom. **3** exhibits Ga–Cl distances of 222.9(2)‐225.2(3) pm and Ga–O distances of 211.2(6) pm and 215.5(5) pm, respectively. The angles Ga–Mo–Ga (82.3(1) °), Cp–Mo–Cp (144.2(1) °), Cl–Ga–Cl (99.6(1), 101.7(1) °), and Mo–Ga–O (109.3(2), 110.4(2) °) are in agreement with the distorted tetrahedral arrangement. Beside single‐crystal structure analysis, FT‐IR spectroscopy and X‐ray powder diffraction (XRD) were performed to validate the structure and purity of **3** (Figure S7, Supporting Information).

Despite the high reactivity of the first reported Tb(0) nanoparticles and the formation of **1‐3** as new compounds, the Tb(0) nanoparticles only serve as a reducing agent and partly react with the ionic liquid. Interestingly, mass spectrometry (MS) nevertheless points to the presence of Tb–Mo–Ga‐ and Tb–Mo‐containing species in solution (e.g., [BMIm] [TbMoCl_7_(Cp)(THF)_3_(MIm)_2_], [BMIm][Tb_2_MoGaCl(Cp)(THF)_3_], [Tb_2_MoCl_3_(THF)_4_], [Tb_2_MoGaCl(THF)(MIm)], [Tb_2_Ga(Cp)_2_], [TbMoCl_2_(Cp)(THF)_2_]; Figures S10–S19, Supporting Information) and confirms the formation of Tb‐containing intermediates for the reaction of Tb(0) nanoparticles and [Cp_2_MoCl_2_]. Although no crystallization of the respective intermediates occured until now, this observation suggests to perform further attempts to crystallize and identify potential compounds in the future. At present, we have also performed comparable reactions with the less reactive V(0) nanoparticles (*E^0^_bulk_* (V/V^3+^) = –0.87 V).^[^ ^7^ ^]^ Similar to the synthesis of **3**, [BMIm]Cl/GaCl_3_ was added with stoichiometric amounts. Since THF showed coordination to Ga^3+^ in **3**, we also replaced THF by toluene as the solvent. With these modified conditions, V(0) nanoparticles were reacted with [Cp_2_MoCl_2_], [BMIm]Cl/GaCl_3_ in toluene at 50 °C, resulting in the formation of moisture‐ and air‐sensitive yellow needles of [BMIm][Cp_2_MoGa_2_Cl_5_] (**4**) with a yield of ≈80 % (in relation to the amount of V(0) nanoparticles). The formation can be rationalized by:

(3) $$\begin{array}{ccc} 4\;V(0) & + & 3\;{\mathrm{Cp}}_{2}{\mathrm{MoCl}}_{2}+6\;{\mathrm{GaCl}}_{3}+3\;\left[\mathrm{BMIm}\right]\mathrm{Cl}\;\\ & & \to \;3\;\left[\mathrm{BMIm}\right]\left[{\mathrm{Cp}}_{2}{\mathrm{MoGa}}_{2}{\mathrm{Cl}}_{5}\right]+4\;{\mathrm{VCl}}_{3} \end{array}$$

Similar to the reaction with terbium, however, vanadium still only serves as a reducing agent without incorporation into the product.

The single‐crystal structure analysis reveals **4** to crystallize in the monoclinic space group *P*2_1_ */c* (Table S1 and Figure S5, Supporting Information) and to consist of [Cp_2_MoGa_2_Cl_5_]^–^ anions and [BMIm]^+^ cations (Figure 4d). Similar to **1**, a central Cp_2_Mo unit is distorted tetrahedrally coordinated by two Ga atoms and two Cp ligands with Mo–Ga distances of 254.1(1) pm (Mo–C: 227.2(7)‐231.5(7) pm). The Ga atoms are also distorted tetrahedrally coordinated by three Cl and one Mo atom. In contrast to **1** and **2**, however, the two Ga centers are here bridged by one Cl atom (Figure 4c). As expected, the Ga–Cl distances of the bridging Cl atom (244.4(2), 253.1(2) pm) are longer than for the non‐bridging Cl atoms (223.7(2)‐225.8(2) pm). Finally, the Ga^…^Ga distance (302.7(1) pm) is beyond the range of Ga–Ga bonding (231.9(3)‐278.7(1) pm).^[^ ^19^, ^20^, ^21^ ^]^ The angles Cp–Mo–Cp (142.7(1) °), Ga–Mo–Ga (73.1(1) °), Cl–Ga–Cl (95.0(1)‐100.5(1) °), and Mo–Ga–Cl (104.4(1)‐126.6(1) °) are in agreement with a distorted tetrahedral coordination of Mo and Ga. FT‐IR spectroscopy was used to validate the composition of **4** (Figure S8, Supporting Information).

In general, Mo–Ga and W–Ga bonds such as observed in **1**‐**4** were yet rarely described. Usually, compounds with Mo–Ga or W–Ga bonds exhibit coordination via the carbon atom of the ligand only (e.g., with Cp, Cp*, CO as ligands; Table 1).^[^ ^22^, ^23^, ^24^, ^25^ ^]^ Only two examples show gallium coordinated by nitrogen (i.e., imidazole, 1,2‐bis[(2,6‐diisopropylphenyl)imino]acenaphthene).^[^ ^26^, ^27^ ^]^ A coordination with more electronegative ligands such as halides is reported to result in poor stability. Thus, only a few compounds with Mo–Ga bonds contain Ga coordinated by halides and/or ethers (Table 1).^[^ ^28^, ^29^ ^]^ The Ga–Mo/Ga–W distances in **1**‐**4** are in agreement with the known examples (Table 1). A redox approach based on reactive metal nanoparticles as well as a sole coordination of gallium with halides for compounds with Mo–Ga/W–Ga bonding are generally reported for the first time and illustrate the potential of the here applied redox approach.

To elucidate the electronic structures of **1‐4**, density functional theory (DFT) computation within the resolution‐of‐the‐identity (RI) was performed.^[^ ^30^ ^]^ One‐component calculations and two‐component calculations were used, including the corresponding relativistic effective core potentials for the Mo and W atoms (see SI). The equilibrium ground‐state geometries were optimized using TURBOMOLE's jobex script within the unrestricted Kohn‐Sham formalism (**Figure** **4**; Table S3, Supporting Information). For [Cp_2_Mo{GaCl_2_(THF)}_2_] (**3**) different structures were investigated. Thus, a structure displaying *C_2_* symmetry with an energy 11.8 kJ mol^−1^ lower than the provided experimental structure with *C_1_* symmetry was obtained. The *C_2v_* point group for [Cp_2_MoGa_2_Cl_5_]^–^, [Cp_2_Mo(GaCl_3_)_2_]^–^, and [Cp_2_W(GaCl_3_)_2_]^–^ as well as the *C_2_* point group for [Cp_2_Mo{GaCl_2_(THF)}_2_] were used throughout all one‐component calculations while the *C_1_* point group was applied for two‐component calculations. However, the two‐component geometry optimizations kept the symmetry of the respective complexes. TURBOMOLE's NumForce script was used to compute harmonic vibrational frequencies, which were all real, therefore confirming that the optimization resulted in a minimum on the potential energy surface. Furthermore, a natural population analysis (NPA)^[^ ^31^ ^]^ was carried out to determine the natural charges of the V, Ga, Mo, and W atoms (Table S3, Supporting Information).^[^ ^32^ ^]^ The highest occupied *d*‐orbital turned out to be singly occupied for [Cp_2_Mo(GaCl_3_)_2_]^–^ and [Cp_2_W(GaCl_3_)_2_]^–^ but doubly occupied for [Cp_2_MoGa_2_Cl_5_]^–^ and [Cp_2_Mo{GaCl_2_(THF)}_2_]. The respective natural molecular orbitals (NMO) of the *d*‐orbital on V, Mo, and W as well as the localized orbitals with contributions from W–Ga or Mo–Ga are visualized in Figures S20 and S21 (Supporting Information).

The [Cp_2_Mo(GaCl_3_)_2_]^–^ and [Cp_2_W(GaCl_3_)_2_]^–^ anions may formally be interpreted to consist of two neutral GaCl_3_ moieties, two Cp^–^ anions, and a Mo^+^ or W^+^ cation, respectively. The cations are 4*d* ^5^ and 5*d* ^5^ systems. The anions show one singly occupied *d*‐orbital (*^2^A_1_* ground state in *C_2v_* symmetry; Figure S20b,d, Supporting Information). The [Cp_2_MoGa_2_Cl_5_]^–^ anion may be seen to consist of a [Ga_2_Cl_5_]^+^ moiety, two Cp^–^ anions, and a formally neutral Mo atom. In sum, this results in a closed‐shell electronic state (*^1^A_1_* ground state in *C_2v_* symmetry). Finally, [Cp_2_Mo{GaCl_2_(THF)}_2_] can be considered to exhibit two neutral THF molecules, two [GaCl_2_]^+^ moieties, two Cp^–^ anions, and a formally neutral Mo atom, which results in a closed‐shell electronic state (*^1^A* ground state in *C_2_* symmetry). The highest doubly occupied *d*‐orbitals of [Cp_2_MoGa_2_Cl_5_]^–^ and [Cp_2_Mo{GaCl_2_(THF)}_2_] are shown in Figure S20a,c (Supporting Information), respectively.

### 2.3 Reactions with Carbonyl Precursors M(CO)_6_ (M: Mo, W)

Since Tb(0) and V(0) nanoparticles in the syntheses of **1**‐**4** only serve as reducing agents without incorporation of the metals into the product, a synproportionation of V(0) nanoparticles with V_2_O_5_ in the presence of the azacrown ether H_4_Cyclal as a ligand and the carbonyls Mo(CO)_6_ or W(CO)_6_ was tested. The reactions were performed at 160 °C in *n*‐dodecane, resulting in deep‐green crystals of [VO(H_2_Cyclal)Mo(CO)_4_] (**5**) and [VO(H_2_Cyclal)W(CO)_4_] (**6**). Both were obtained with ≈60% yield according to the following equation:

(4) $$\begin{array}{ccc} 3\;V(0) & + & 5\;H_{4}\mathrm{Cyclal}+5\;M{\left(\mathrm{CO}\right)}_{6}+V_{2}O_{5}\;\\ & & \to \;5\;\left[\mathrm{VO}\left(H_{2}\mathrm{Cyclal}\right)M{\left(\mathrm{CO}\right)}_{4}\right]+5\;H_{2}+10\;\mathrm{CO} \end{array}$$

(5) (M:Mo,W)

**Figure 5:**
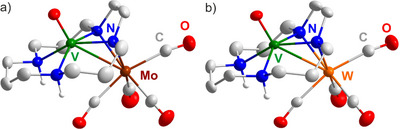
Structures of a) [VO(H_2_Cyclal)Mo(CO)_4_] (**5**), b) [VO(H_2_Cyclal)W(CO)_4_] (**6**) (H atoms only partially shown for clarity).

Single‐crystal structure analysis revealed **5** and **6** to crystallize in the monoclinic space group *P*2_1_ */n* (Table and Figure S6, Supporting Information). The title compounds consist of a vanadyl unit coordinated by a partially deprotonated [H_2_Cyclal]^2–^ ligand and an additional Mo(CO)_4_ or W(CO)_4_ unit (**Figure** **5**). The V–Mo distance in **5** is 294.2(1) pm, and the V–W distance in **6** is 293.8(1) pm. Both are also bridged by deprotonated amine groups of the azacrown ether. The V–O distances are 161.1(1) pm (**5**) and 161.0(2) pm (**6**). The V–N distances of protonated amine groups (**5**: 215.9(2)‐216.0(2) pm; **6**: 215.3(2)‐215.5(2) pm), as expected, are significantly longer than those distances of deprotonated, bridging amine groups (**5**: 202.8(2)‐203.3(2) pm; **6**: 203.6(2) pm). The protonation of the non‐bridging amine groups is additionally confirmed by V–N–C angles of 112.1(1)‐113.3(1) ° (**5**) and 112.0(2)‐113.6(2) ° (**6**), which point to a tetrahedral arrangement (Figure 5). Furthermore, **5** exhibits Mo–N distances of 227.5(2) and 227.9(2) pm and V–N–Mo angles of 85.8(1) and 86.1(1) ° for the bridging amine groups. **6** exhibits W–N distances of 225.3(2) and 225.9(2) pm with V–N–W angles of 86.2(1) and 86.3(1) °. Finally, Mo and W are each coordinated by four CO ligands (Mo–C: 197.2(3)‐207.6(2) pm; W–C: 196.6(3)‐205.7(3) pm). CO ligands in *trans*‐position to the bridging N atoms show almost linear coordination (Mo–C–O: 177.1(2), 178.9(2) °; W–C–O: 176.7(3), 178.1(2) °), whereas CO ligands in *cis*‐position show certain bending (Mo–C–O: 170.5(2), 170.6(2) °; W–C–O: 171.2(3), 171.8(2) °), which can be attributed due to the steric demand of the azacrown ether (Figure 5).

**Figure 6:**
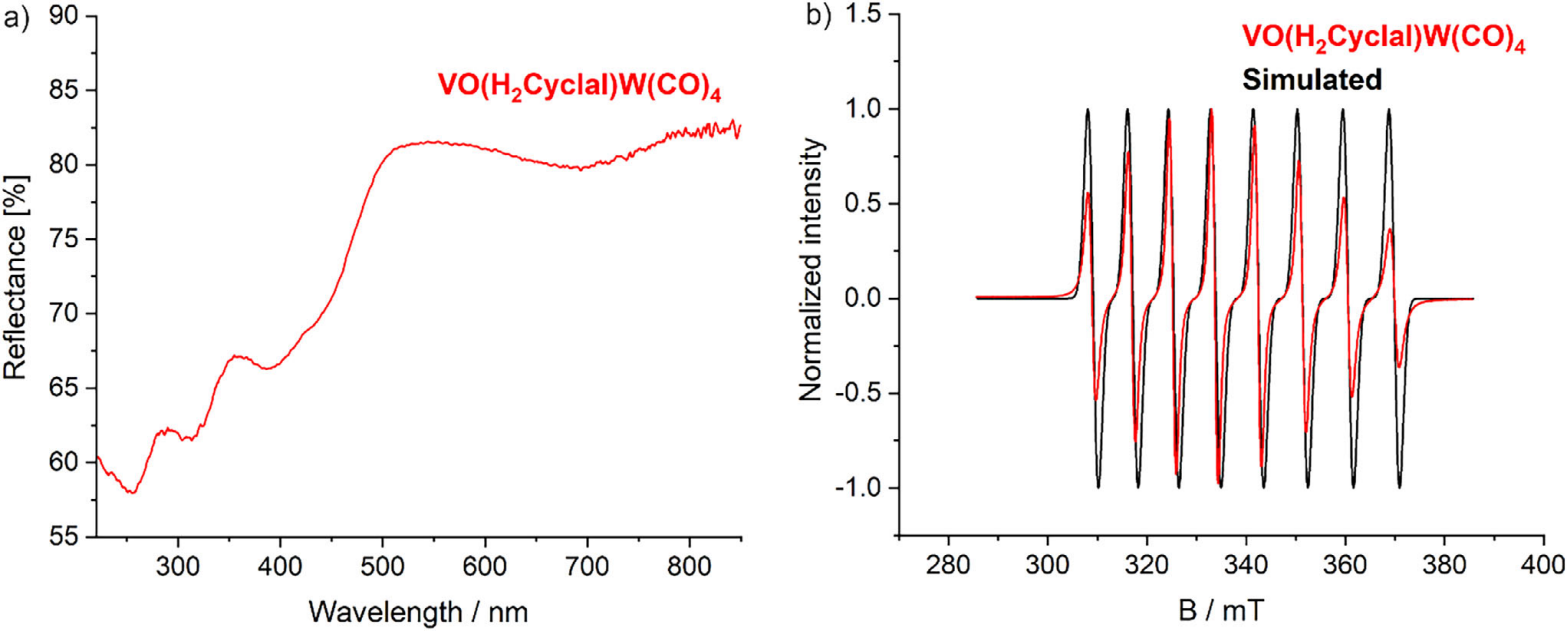
UV–vis spectrum a) and EPR spectrum b) of [VO(H_2_Cyclal)W(CO)_4_] (**6**).

As **5** and **6** are chemically and structurally very similar, a detailed examination of structure and bonding is focused on [VO(H_2_Cyclal)W(CO)_4_] (**6**). First of all, FT‐IR spectra confirm the presence of the azacrown‐ether and of CO (Figure S9b, Supporting Information). X‐ray powder diffraction (XRD) with Rietveld refinement validates the crystal structure and the purity of the compound (Figure S9a, Supporting Information). UV–vis spectra point to absorptions below 500 nm, which are in agreement with the deep green color of **6** (**Figure** **6**a). Electron‐spin resonance (EPR) spectroscopy was performed with a solution of **6** in CH_2_Cl_2_ to determine the oxidation states of vanadium and tungsten (Figure 6b). The EPR spectrum shows an eight‐line signal, which is in agreement with one unpaired electron coupled to V(IV) (^51^V, *I* = 7/2). This, *vice versa* requests an oxidation state of W(0).

**Figure 7:**
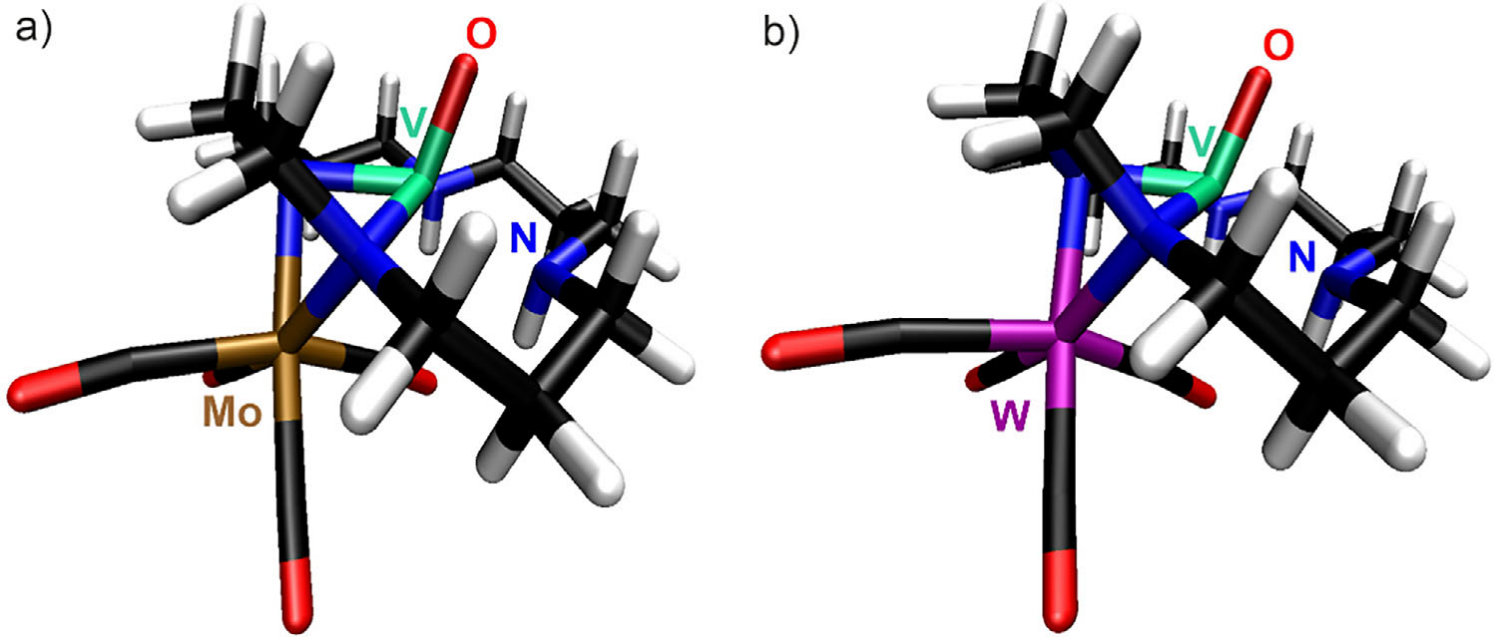
Optimized equilibrium geometries at the B3LYP/dhf‐TZVP‐2c level for a) [VO(H_2_Cyclal)Mo(CO)_4_] (**5**) and b) [VO(H_2_Cyclal)W(CO)_4_] (**6**).

The electronic structure of [VO(H_2_Cyclal)Mo(CO)_4_] (**5**) and [VO(H_2_Cyclal)W(CO)_4_] (**6**) was also examined by DFT calculation (see SI),^[^ ^30^ ^]^ including the corresponding relativistic effective core potentials for the Mo and W atoms. The *C_2_* point group was used throughout for all one‐component calculations of **5** and **6** while the *C_1_* point group was applied for two‐component calculations. Furthermore, a natural population analysis^[^ ^31^ ^]^ was carried out to determine the natural charges of the V, Mo, and W atoms (**Figure** **7**; Table S3, Supporting Information). NMOs were calculated at the B3LYP/dhf‐TZVP‐2c level of theory and localized using the Pipek‐Mezey method.^[^ ^32^ ^]^ The highest occupied *d*‐orbital was shown to be singly occupied for [VO(H_2_Cyclal)Mo(CO)_4_] and [VO(H_2_Cyclal)W(CO)_4_] (Figure 7; Table S3 and Figure S20, Supporting Information). The localization did not result in localized orbitals with contributions from both V and *M* (*M*: Mo, W), thus excluding a V–M bond. [VO(H_2_Cyclal)Mo(CO)_4_] and [VO(H_2_Cyclal)W(CO)_4_] may be viewed as Mo or W complexes with a [VO(H_2_Cyclal)] moiety serving as a bidentate ligand comparable to ethylenediamine (en) in [*M*(CO)_4_(en)] (*M*: Mo, W), which are complexes with closed‐shell, singlet ground states.^[^ ^33^ ^]^ The calculated V–Mo distance in **5** (CAM‐B3LYP: 294.2 pm; Table S3, Supporting Information) and V–W distance in **6** (CAM‐B3LYP: 294.2 pm; Table S3, Supporting Information), is well in agreement with the experimental data (V–Mo in **5**: 294.2(1) pm; V–W in **6**: 293.8(1) pm). The [VO(H_2_Cyclal)] moiety in these complexes may be formally considered to consist of (H_2_Cyclal)^2–^, O^2–^, and V^4+^. The V^4+^ cation has a 3*d* ^1^ occupation (Figure S20e,f, Supporting Information).

Compounds with V–W interaction are rare until now. To the best of our knowledge, only two structurally related compounds have been described with a carbyne‐bridged V–W bonding of V(III) and W(I).^[^ ^34^ ^]^ The V–W distance (299.4 pm) in these compounds compares to **6** (293.8(1) pm). EPR spectroscopy also showed a signal of eight‐lines similar to **6**. However, [Et_4_N]_2_[VO(ema)W(CO)_4_] (ema: N,N’‐ethylenebis{2‐mercaptoacetamide}) was described, for which a V–W bond was excluded.^[^ ^4^, ^35^ ^]^ The V–W distance with 329.8 pm is significantly longer in [Et_4_N]_2_[VO(ema)W(CO)_4_] than in **6**, but the oxidation states were described with V(IV) and W(0), as in **6**.

## 3 Conclusion

Nanoparticles of the zerovalent metals terbium and vanadium are prepared, characterized, and examined in regard of their reactivity and reactions. Specifically, they are used as starting materials for the synthesis of new compounds with metal‐metal bonding and/or low‐valent atoms via a redox approach in the liquid phase. The Tb(0) nanoparticles and V(0) nanoparticles are prepared by reduction of TbCl_3_ or VCl_3_ with lithium naphthalenide in THF at room temperature. The metal nanoparticles exhibit small sizes with 2.8 ± 0.4 nm for Tb(0) and 1.2±0.2 nm for V(0). They are colloidally and chemically stable as suspensions in THF or toluene under inert conditions. They are highly reactive when in contact to air, water, or other oxidizing agents, resulting in immediate combustion and/or explosion. The reaction with air or water already indicates as significantly higher reactivity for the nanosized vanadium and terbium, which is comparable to bulk sodium and bulk cesium and, thus, considerably higher as for bulk vanadium and bulk terbium.

The reactivity of the Tb(0) and V(0) nanoparticles is probed for the first time with cyclopentadienyl precursors [Cp_2_ *M*Cl_2_] and carbonyl precursors [*M*(CO)_6_] (*M* = Mo, W). As a result, the new compounds [BMIm][Cp_2_Mo(GaCl_3_)_2_] (**1**), [BMIm][Cp_2_W(GaCl_3_)_2_] (**2**), [Cp_2_Mo{GaCl_2_(THF)}_2_] (**3**), [BMIm][Cp_2_MoGa_2_Cl_5_] (**4**), [VO(H_2_Cyclal)Mo(CO)_4_] (**5**) and [VO(H_2_Cyclal)W(CO)_4_] (**6**) are realized in the liquid phase (THF, toluene, *n*‐dodecane, ionic liquid). The title compounds contain metal‐metal bonding (Mo–Ga, W–Ga) and/or low‐valence metals (Mo(0/I), W(0/I), Ga(III)). Although “only” serving as a reducing agent, the most reactive terbium nanoparticles result in different products as, for instance, reactions with bulk sodium. Interestingly, mass spectrometry also points to the presence of Tb–Mo–Ga‐ and Tb–Mo‐containing species in solution. In sum, the redox approach using Tb(0) and V(0) nanoparticles as starting materials with different reactivity can be a promising option for chemical reactions and the formation of new compounds with metal‐metal bonding or low valence states, utilizing the much higher reactivity of nanosized metals in comparision to the respective bulk metals.

## 4 Experimental Section

### Synthesis Procedures

Details related to the synthesis of the metal nanoparticles and the compounds **1‐6** as well as further details related to their characterization are described in the Supporting Information.

### Analytical Equipment

Details related to analytical techniques and crystallographic data of compounds **1‐6** are described in the Supporting Information.

## Conflict of Interest

The authors declare no conflict of interest.

## Supporting information



Supporting Information

Supplemental Video 1

## Data Availability

The data that support the findings of this study are available from the corresponding author upon reasonable request.
