# Supplementary material for: Terbium and Vanadium Metal Nanoparticles Reactive Starting Materials for Liquid‐Phase Syntheses
Source: Small. 2025 Jun 9;21(31):2503498. doi: 10.1002/smll.202503498 (PMC12332814; doi:10.1002/smll.202503498)
Supplement: Supplementary file 1 — Supporting Information [file SMLL-21-2503498-s002.docx]

**Supporting Information (SI)**

**Terbium and Vanadium Metal Nanoparticles:**

**Reactive Starting Materials for Liquid-Phase Syntheses**

**Andreas Reiß^[a]^, Anja Appenzeller^[b]^, Jule J. Baur^[a]^, Jonas O. Wenzel^[a]^, Radian Popescu^[c]^, Kathrin Beuthert^[d]^, Stefanie Dehnen^[d]^, Yolita M. Eggeler^[c]^, Frank Breher^[a]^, Wim Klopper^[b]^*****, and Claus Feldmann^[a]^***

^[a]^ *Dr. A. Reiß, B.Sc. J. Baur, Dr. J. O. Wenzel, Prof. Dr. F. Breher, Prof. Dr. C. Feldmann*

*Institute for Inorganic Chemistry, Karlsruhe Institute of Technology (KIT)*

*Engesserstrasse 15, D-76131 Karlsruhe, Germany*

[*claus.feldmann@kit.edu*](mailto:claus.feldmann@kit.edu)

^[b]^ *M.Sc. A. Appenzeller, Prof. Dr. W. Klopper*

*Institute of Physical Chemistry, Karlsruhe Institute of Technology (KIT),*

*Fritz-Haber-Weg 2, D-76131 Karlsruhe, Germany*

*klopper@kit.edu*

^[c]^ *Dr. R. Popescu, Prof. Dr. Y. M. Eggeler*

*Laboratory for Electron Microscopy, Karlsruhe Institute of Technology (KIT)*

*Engesserstrasse 7, D-76131 Karlsruhe, Germany*

^[d]^ *Dr. K. Beuthert, Prof. Dr. S. Dehnen*

*Institute of Nanotechnology, Karlsruhe Institute of Technology (KIT),*

*Hermann-von-Helmholtz-Platz 1, D-76344 Eggenstein-Leopoldshafen, Germany*

**Content**

**1. Analytical Techniques**

**2. Synthesis of Tb(0)/V(0) Nanoparticles and Compounds 1-6**

**3. Characterization of Tb(0)/V(0) Nanoparticles**

**4. Crystallographic Data of Compounds 1-6**

**5. Characterization of Compounds 1-6**

**6. Computation**

**7. References**

**1. Analytical Techniques**

**Transmission electron microscopy (TEM).** For the TEM sample preparation, diluted suspensions of as-prepared Tb(0)/V(0) nanoparticles in THF were deposited on a commercial 400 μm mesh Cu-grid (Plano) covered by a holey amorphous carbon film with a nominal thickness of 3 nm. After deposition, TEM grids are dried to evaporate the solvent. To avoid any oxidation during TEM sample preparation, the nanoparticle deposition was performed under argon atmosphere in a glovebox. Thereafter, the grids were transferred with a suitable vacuum/inert gas transfer module (GATAN) into the transmission electron microscope without any contact to air. TEM and high-resolution (HR) TEM were conducted with an aberration-corrected FEI Titan^3^ 80-300 operated at 300 keV electron energy. HRTEM images were evaluated by calculating the two-dimensional Fourier transform (FT), which yields information on the crystal structure (lattice parameters and crystal symmetry) of single nanoparticles. The analysis was performed by comparing the experimental FT and calculated diffraction patterns with Miller indices, where the latter were obtained by using the Jems (Java version of the electron microscopy simulation) software.^[S1]^

**X-ray powder diffraction (PXRD)** was performed on a STOE STADI-MP diffractometer operating with Cu-K_α1_-radiation (λ = 154.0598 pm) monochromated by a focusing Ge crystal. Powder samples were diluted with an equal amount of dried glass spheres (9-13 μm, Sigma-Aldrich) to reduce the X-ray absorption of the nanoparticles and filled into glass capillaries (0.4 mm in diameter, Hilgenberg) under argon. Rietveld refinements were performed with the program TOPAS-Academic (Version 5), using the cif-data to investigate the phase purity of the title compound. The refinement was carried out by a simple axial model.

**Fourier-transform infrared spectroscopy.** FT-IR spectroscopy was performed on a Vertex 70, equipped with a Platinum A 225 ATR unit with an air-tight sample chamber (Bruker) and on a Alpha II platinum ATR (Bruker) located in a glovebox. Obtained infrared spectra of the Tb(0)/V(0) nanoparticles and the title compounds **1**-**6** were analyzed using the OPUS program.

**Elemental analysis (C/H/N analysis)** of the as-prepared Tb(0)/V(0) nanoparticles was performed on an Vario Micro Cube (Elementar) via complete thermal combustion of the respective sample in an atmosphere of pure oxygen at a temperature of 1,100 °C.

**Crystal structure determination and refinement.** For **s**inge-crystal X-ray diffraction, suitable single crystals of the title compounds **1**-**6** were manually selected, covered by inert oil (perfluoropolyalkylether, ABCR) and mounted onto a micro gripper (MiTeGen). For compound **1**, data collection was performed at 100 K on an StadiVari Diffractometer with Euler geometry (Stoe) using Mo-K_α_ radiation (λ = 7.1073 Å, graphite monochromator). For compounds **2**-**6**, data collection was performed at 180 K on an IPDS II image plate diffractometer (Stoe, Darmstadt) utilizing monochromatized Mo-K_α1_ radiation (λ = 71.073 pm, graphite monochromator). Data reduction and multi-scan absorption correction were performed using the X-AREA software package and Stoe LANA (version 1.75).^[S2]^ Space group determination based on systematic absence of reflections was performed by XPREP. For structure solution and refinement, SHELXT and SHELXL were used.^[S3]^ All non-hydrogen atoms were refined anisotropically. Detailed information on crystal data and structure refinement are listed in Table S1 and S2. DIAMOND was used for all illustrations.^[S4]^ Further details related to the crystal structures may be obtained from the joint CCDC/FIZ Karlsruhe deposition service on quoting the depository numbers 2417222-2417227.

**UV-Vis spectroscopy** was conducted using a UV-2700 spectrometer (Shimadzu), equipped with an integrating sphere in the wavelength interval of 220-850 nm against BaSO_4_ as a reference. For measurement, about 0.5 mg of the title compound **5** was mortared with 50 mg of BaSO_4_ and the resulting powder was placed in a suitable, airtight sample holder under inert conditions.

**Continuous-wave electron paramagnetic resonance (CW-EPR) spectroscopy.** CW-EPR measurements (X-band) were performed with a Bruker EMXplus spectrometer (Bruker, Germany). Field calibration was performed using 2,2-diphenyl-1-picrylhydrazyl (DPPH) as reference with a g-value of 2.0036.^[S5]^ For the measurement, a 1 mM solution of compound **5** in DCM was prepared in a glovebox and placed in a quartz glass tube which was sealed airtight. The experimental spectrum was simulated by using the EasySpin 5.2.35 implementation in MatLab R2023b.

**Mass spectrometry** was performed with a freshly prepared solution of [VO(H_2_Cyclal)W(CO)_4_] **(6)** in acetonitrile with a Thermo Fischer Scientific Finnigan LTQ-FT spectrometer in the negative ion mode. The sample was prepared inside of a glove box, where it was dissolved in anhydrous acetonitrile, stirred to dissolve, and finally filtered through a Teflon syringe filter with a pore size of 0.70 μm. This solution was injected into the spectrometer with a gastight 250 µL Hamilton syringe by syringe pump infusion. All capillaries within the system were washed with dry acetonitrile for 30 minutes prior to the measurement to avoid decomposition. Measurements were performed with the following ESI parameters: spray voltage 3.6 kV, capillary temperature 300 °C, capillary voltage –12, tube lens voltage –86.7, sheath gas 45, sweep gas 0, auxiliary gas 40.

**2. Synthesis of Tb(0)/V(0) Nanoparticles and Compounds 1-6**

**General.** All sample handling and reactions were performed under argon atmosphere using standard Schlenk techniques and gloveboxes (MBraun Unilab, O_2_/H_2_O < 1 ppm). All glassware was evacuated (*p* ≤ 10^–3^ mbar), heated, and flushed with argon three times prior to use to remove all residual moisture.

**Chemicals.** Tetrahydrofurane (THF, Seulberger, 99 %) and toluene (Tol, Seulberger, 99 %) were refluxed over sodium with benzophenone and destilled prior to use. n-Dodecane (Sigma-Aldrich, ≥ 99 %) was refluxed under reduced pressure (p ≤ 10^–3^ mbar) over sodium and destilled prior to use. Lithium metal (AlfaAesar, 99 %) as well as sodium metal (Riedel-de-Haën, 99 %) were freshly cut in a glovebox under argon atmosphere prior to use. 1-Butyl-3-methylimidazoliumchloride ([BMIm]Cl, IoLiTec, 99 %) was dried under reduced pressure (p ≤ 10^–3^ mbar) at 130 °C for 48 h. TbCl_3_ (Sigma-Aldrich, 99.99 %), VCl_3_ (Alfa Aesar, 99 %), naphthalene (Alfa Aesar, 99 %), 1,4,8,12-tetraazacyclopentadecane (H_4_Cyclal, ABCR, 98 %), W(CO)_6_ (ABCR, 99 %), Mo(CO)_6_ (Sigma-Aldrich, 98 %), V_2_O_5_ (Riedel-de-Haën, > 99 %), Cp_2_WCl_2_ (ABCR, 97 %), Cp_2_MoCl_2_ (ABCR, 98 %), and GaCl_3_ (ABCR, 99.99 %) were used as received.

**Tb(0) nanoparticles.** 6.9 mg of lithium (1.00 mmol), 135.0 mg of naphthalene (1.05 mmol), and 87.5 mg of TbCl_3_ (0.33 mmol) were stirred in 15 mL of THF over a period of 12 hours. The resulting deep-black suspension of Tb(0) nanoparticles was centrifuged (22,000 rpm, 42,500 × g) and the as-prepared nanoparticles were then purified by washing twice with 15 mL of THF to remove excess starting materials, naphthalene and LiCl. Thereafter, the Tb(0) nanoparticles were dried in vacuum (*p* ≤ 10^–3^ mbar, 15 min) to obtain powder samples.

**V(0) nanoparticles.** V(0) nanoparticles were prepared following a previously published procedure of our group.^1^ Thus, 46.0 mg of sodium (2.00 mmol), 270.0 mg of naphthalene (2.10 mmol), and 105.4 mg of VCl_3_ (0.67 mmol) were stirred in 15 mL of THF over a period of 12 hours. The resulting deep-black suspension of V(0) nanoparticles was centrifuged (22,000 rpm, 42,500 × g). The obtained V(0) nanoparticles were then purified by washing twice with 15 mL of THF to remove excess starting materials and naphthalene. The as-prepared V(0) nanoparticles were dried in vacuum (*p* ≤ 10^–3^ mbar, 15 min) to obtain powder samples.

**[BMIm][Cp_2_Mo(GaCl_3_)_2_] (1).** 40.9 mg of dried Tb(0) nanoparticles (0.26 mmol), 76.5 mg of Cp_2_MoCl_2_ (0.26 mmol), 200.0 mg of [BMIm]Cl (1.14 mmol), and 260.0 mg of GaCl_3_ (1.48 mmol) were sealed in a glass ampoule. After heating the ampoule to 50 °C for 4 days and subsequent cooling to room temperature with a rate of 1 K/h, colourless crystals of **1** were obtained with a yield of about 40 % in relation to the used amount of Tb(0) nanoparticles.

**[BMIm][Cp_2_W(GaCl_3_)_2_] (2).** 40.9 mg of dried Tb(0) nanoparticles (0.26 mmol), 100.1 mg of Cp_2_WCl_2_ (0.26 mmol), 200.0 mg of [BMIm]Cl (1.14 mmol), and 260.0 mg of GaCl_3_ (1.48 mmol) were sealed in a glass ampoule and heated to 50 °C for 4 days. After cooling to room temperature with a rate of 1 K/h, colourless crystals of **2** were obtained with a yield of about 40 % in relation to the used amount of Tb(0) nanoparticles.

**[Cp_2_Mo(GaCl_2_(THF))_2_] (3).** 40.9 mg of dried Tb(0) nanoparticles (0.26 mmol) were reacted in a Schlenk flask with 57.7 mg of Cp_2_MoCl_2_ (0.19 mmol), 68.3 mg of GaCl_3_ (0.39 mmol), and 33.9 mg of [BMIm]Cl (0.19 mmol) in 0.3 mL of THF. After 4 days at room temperature, yellow-brown crystals of **3** were obtained with quantitative yield.

**[BMIm][Cp_2_MoGa_2_Cl_5_]** **(4).** 25.7 mg of dried V(0) nanoparticles (0.51 mmol), 75.0 mg of Cp_2_MoCl_2_ (0.25 mmol), 88.9 mg of GaCl_3_ (0.51 mmol), 44.2 mg of [BMIm]Cl (0.25 mmol), and 0.5 mL of Tol were sealed in a glass ampoule. The ampoule was heated to 50 °C for 7 days. After natural cooling to room temperature, yellow needles of **4** were obtained with a yield of about 80 % in relation to the used amount of Cp_2_MoCl_2_.

**[VO(H_2_Cyclal)Mo(CO)_4_] (5).** 25.7 mg of dried V(0) nanoparticles (0.51 mmol), 108.2 mg of H_4_Cyclal (0.51 mmol), 133.3 mg of Mo(CO)_6_ (0.51 mmol), 41.4 mg of V_2_O_5_ (0.23 mmol), and 1 mL of *n*-dodecane were sealed in a glass ampoule and heated to 160 °C for 4 days. After cooling to room temperature with a rate of 2 K/h, green crystals of **6** were obtained with a yield of about 50 % in relation to the used amount of V(0) nanoparticles.

**[VO(H_2_Cyclal)W(CO)_4_] (6).** 25.7 mg of dried V(0) nanoparticles (0.51 mmol), 108.2 mg of H_4_Cyclal (0.51 mmol), 177.7 mg of W(CO)_6_ (0.51 mmol), 41.4 mg of V_2_O_5_ (0.23 mmol), and 2.5 mL of *n*-dodecane were sealed into a glass ampoule and heated to 160 °C for 4 days. After cooling to room temperature with a rate of 2 K/h, green crystals of **5** were obtained with a yield of about 60 % in relation to the used amount of V(0) nanoparticles.

**3. Characterization of Tb(0)/V(0) Nanoparticles**

In addition to electron microscopy (*see main paper: Figure 2*), particle size and size distribution were exemplarily also determined by dynamic light scattering (DLS) for the as-prepared V(0) nanoparticles in THF suspension (Figure S1). DLS analysis is generally complicated by two issues. First of all, light-scattering analysis of the deep black suspensions is hampered by their strong absorption even after dilution. As the DLS instrument was not located inside of a glovebox, moreover, the cuvettes containing the nanoparticle suspension needed to be handled outside the glovebox with cuvettes of limited tightness to air (i.e. cuvettes cannot be evacuated and heated to remove all traces of water). This was also the reason to exemplarily use V(0) nanoparticles instead of the even more reactive Tb(0) nanoparticles. Surprisingly, DLS analysis shows two peaks (Figure S1). The peak at 2.4±0.4 nm is well in agreement with TEM data (1.2±0.2 nm) and reflects the hydrodynamic diameter of the V(0) nanoparticles with THF adsorbed on the particle surface. The peak at 5.7±0.7 nm represents the doubled hydrodynamic diameter. As both peaks are narrow and as no particles were detected at larger sizes, an agglomeration of the V(0) nanoparticles did not occur. The observation of the doubled hydrodynamic diameter can be ascribed to pairs of V(0) nanoparticles, which might originate from weak magnetic interaction of the small-sized particles.


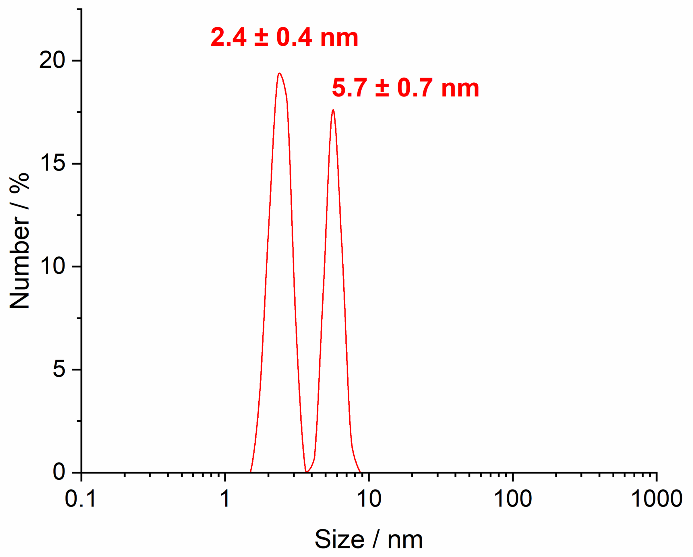


**Figure S1.** DLS analysis of the as-prepared V(0) nanoparticles in THF suspension.

Fourier-transform infrared (FT-IR) spectra indicate the surface functionalization of the as-prepared Tb(0) nanoparticles and V(0) nanoparticles. Thus, vibrations such as *ν*(C–H): 3000-2800 cm^–1^ and *ν*(C‑O): 1050-800 cm^–1^ point to the presence of THF (Figure S2). Furthermore, a series of sharp vibrations at 1600-800 cm^–1^ relate to naphthalene. Together with elemental analysis (*see main paper*), the Tb(0)/V(0) nanoparticles are predominately functionalized by THF with a minor contribution of naphthalene.

**
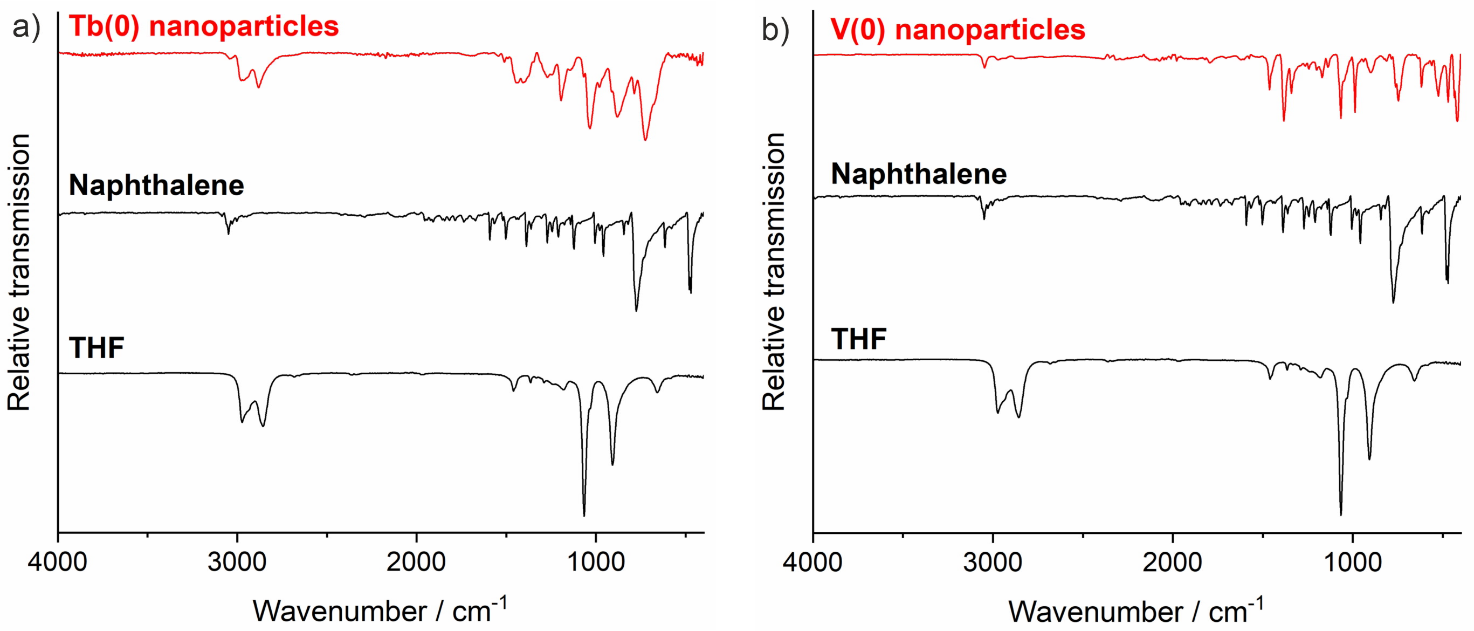
**

**Figure S2.** FT-IR spectra of the as-prepared Tb(0) (a) and V(0) (b) nanoparticles (with spectra of naphthalene and THF as references).

**4. Crystallographic Data of Compounds 1-6**

Crystallographic and refinement details of the title compounds **1**-**4** are summarized in Table S1, the unit cells of the compounds **1**-**4** are shown in Figure S3-S5. The crystallographic and refinement details of compounds **5** and **6** are summarized in Table S2, the unit cells of compounds **5** and **6** are shown in Figure S6.

**Table S1.** Crystallographic and refinement details of the title compounds **1**-**4**.

| **Data** | **[BMIm][Cp_2_Mo(GaCl_3_)_2_]**  **(1)** | **[BMIm][Cp_2_W(GaCl_3_)_2_]**  **(2)** | **[Cp_2_Mo(GaCl_2_(THF))_2_]**  **(3)** | **[BMIm][Cp_2_MoGa_2_Cl_5_]**  **(4)** |
| --- | --- | --- | --- | --- |
| Sum formula | C_18_H_25_Cl_6_Ga_2_N_2_Mo | C_18_H_25_Cl_6_Ga_2_N_2_W | C_18_H_26_Cl_4_Ga_2_O_2_Mo | C_18_H_25_Cl_5_Ga_2_N_2_Mo |
| Crystal system | Monoclinic | Monoclinic | Tetragonal | Monoclinic |
| Space group | *P*2_1_*/n* | *P*2_1_*/n* | *P*4_1_ (Flack parameter: 0.01(1)) | *P*2_1_*/c* |
| Lattice parameters |  |  |  |  |
| *a* /pm | 751.7(1) | 759.1(1) | 901.6(1) | 1209.3(3) |
| *b* /pm | 2460.6(2) | 2512.3(5) | 901.6(1) | 777.5(1) |
| *c* /pm | 1361.7(1) | 1367.8(3) | 287.8(1) | 2618.0(4) |
| *α* /° | 90 | 90 | 90 | 90 |
| *β* /° | 94.45(1) | 94.39(2) | 90 | 97.07(1) |
| *γ* /° | 90 | 90 | 90 | 90 |
| Cell volume /×10^6^ pm^3^ | *V* = 2510.9(2) | *V* = 2600.7(10) | *V* = 2339.5(5) | *V* = 2442.8(6) |
| Formula units per cell | *Z* = 4 | *Z* = 4 | *Z* = 4 | *Z* = 4 |
| Calculated density /g/cm^3^ | *ρ* = 1.898 | *ρ* = 2.057 | *ρ* = 1.850 | *ρ* = 1.854 |
| Measurement limits | -8 ≤ h ≤ 10  -31 ≤ k ≤ 33  -17 ≤ l ≤ 18 | -9 ≤ h ≤ 9  -29 ≤ k ≤ 29  -16 ≤ l ≤ 13 | -12 ≤ h ≤ 12  -10 ≤ k ≤ 12  -32 ≤ l ≤ 39 | -14 ≤ h ≤ 14  -9 ≤ k ≤ 9  -30 ≤ l ≤ 30 |
| Theta range for data collection | 1.71 to 30.30 ° | 1.62 to 25.00 | 2.26 to 29.17 | 1.70 to 25.00 |
| Linear absorption coefficient /mm^-1^ | *µ* = 3.271 | *µ* = 7.095 | *µ* = 3.283 | *µ* = 3.251 |
| Number of reflections  thereof independent | 17772  6279 | 13867  4560 | 13793  5916 | 8202  4296 |
| Refinement method | Full-matrix least-squares on F^2^ for all | | | |
| Merging | *R_int_* = 0.029 | *R_int_* = 0.076 | *R_int_* = 0.028 | *R_int_* = 0.134 |
| Number of parameters | 264 | 264 | 262 | 256 |
| Residual electron density  /e^–^·10^-6^ pm^-3^ | 0.83 to -0.52 | 0.99 to -1.22 | 0.52 to -0.40 | 0.58 to -1.22 |
| *R1* (*I* ≥ 2*σ_I_*) | 0.026 | 0.028 | 0.035 | 0.039 |
| *R1* (all data) | 0.043 | 0.055 | 0.041 | 0.140 |
| *wR2* (all data) | 0.054 | 0.054 | 0.098 | 0.062 |
| GooF | 0.959 | 0.789 | 1.076 | 0.713 |

**Table S2.** Crystallographic and refinement details of the title compounds **5** and **6**.

| **Data** | **[VO(H_2_Cyclal)Mo(CO)_4_] (5)** | **[VO(H_2_Cyclal)W(CO)_4_] (6)** | |
| --- | --- | --- | --- |
| Sum formula | C_15_H_24_N_4_O_5_VMo | C_15_H_24_N_4_O_5_VW | |
| Crystal system | Monoclinic | Monoclinic | |
| Space group | *P*2_1_*/n* | *P*2_1_*/n* | |
| Lattice parameters |  |  | |
| *a* /pm | 1162.7(1) | 1156.4(1) | |
| *b* /pm | 1124.7(1) | 1120.9(1) | |
| *c* /pm | 1497.4(2) | 1495.4(1) | |
| *α* /° | 90 | 90 | |
| *β* /° | 90.13(1) | 89.997(7) | |
| *γ* /° | 90 | 90 | |
| Cell volume / ×10^6^ pm^3^ | *V* = 1958.0(4) | *V* = 1938.3(3) | |
| Formula units per cell | *Z* = 4 | *Z* = 4 | |
| Calculated density / g/cm^3^ | *ρ* = 1.653 | *ρ* = 1.971 | |
| Measurement limits | -15 ≤ h ≤ 15  -15 ≤ k ≤ 13  -20 ≤ l ≤ 19 | -15 ≤ h ≤ 14  -15 ≤ k ≤ 15  -20 ≤ l ≤ 15 | |
| Theta range for data collection | 2.22 to 29.22 ° | 2.23 to 29.15 ° | |
| Linear absorption coefficient /mm^-1^ | *µ* = 1.150 | *µ* = 6.444 | |
| Number of reflections  thereof independent | 13904  5255 | 11822  5085 | |
| Refinement method | Full-matrix least-squares on F^2^ for both | |  |
| Merging | *R_int_* = 0.045 | *R_int_* = 0.038 | |
| Number of parameters | 235 | 235 | |
| Residual electron density / e^–^·10^-6^ pm^-3^ | 0.44 to -0.26 | 0.97 to -1.41 | |
| *R1* (*I* ≥ 2*σ_I_*) | 0.0264 | 0.021 | |
| *R1* (all data) | 0.044 | 0.023 | |
| *wR2* (all data) | 0.057 | 0.056 | |
| GooF | 0.889 | 1.073 | |

**
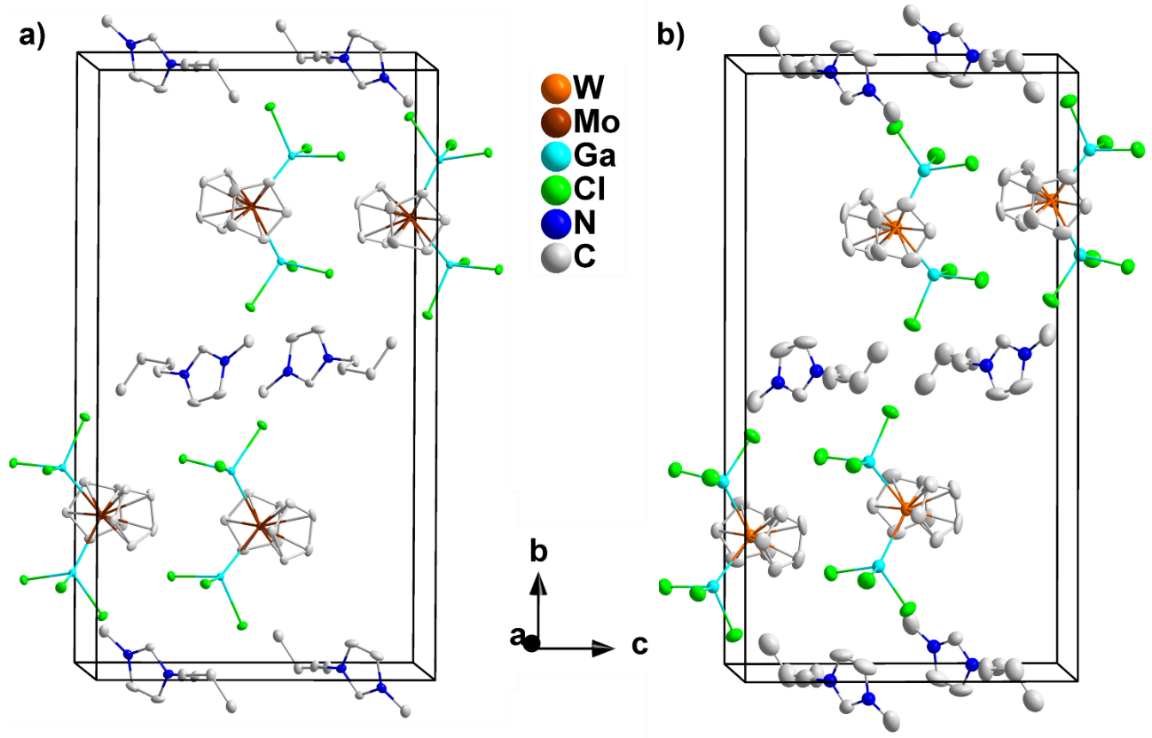
**

**Figure S3.** Unit cells of a) [BMIm][Cp_2_Mo(GaCl_3_)_2_] (**1**), b) [BMIm][Cp_2_W(GaCl_3_)_2_] (**2**) (H atoms not shown for clarity).

**
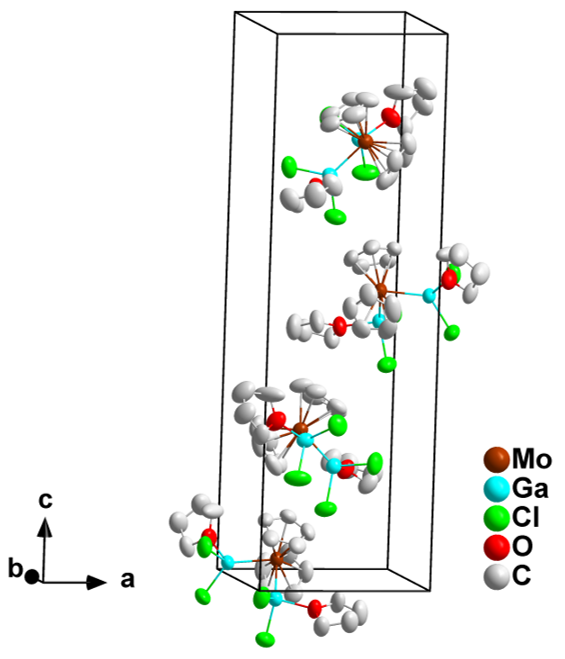
**

**Figure S4.** Unit cell of [Cp_2_Mo(GaCl_2_(THF))_2_] (**3**) (disorder and H atoms not shown for clarity).


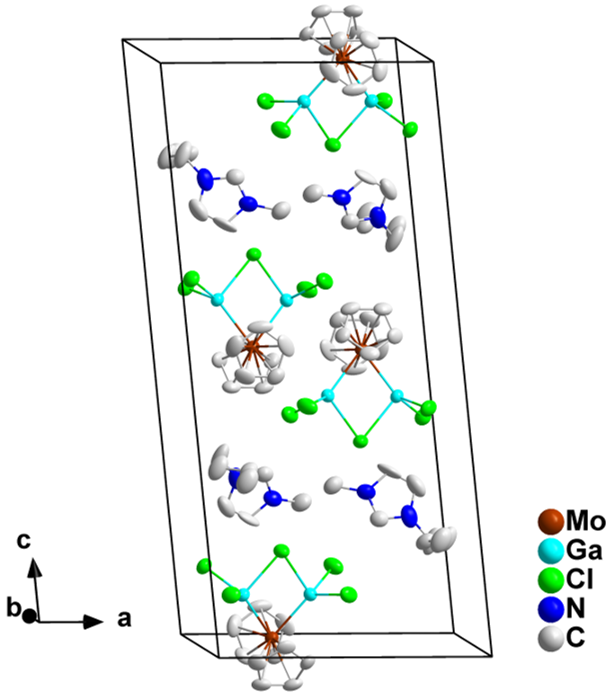


**Figure S5.** Unit cell of [BMIm][Cp_2_MoGa_2_Cl_5_] (**4**) (H atoms not shown for clarity).

**
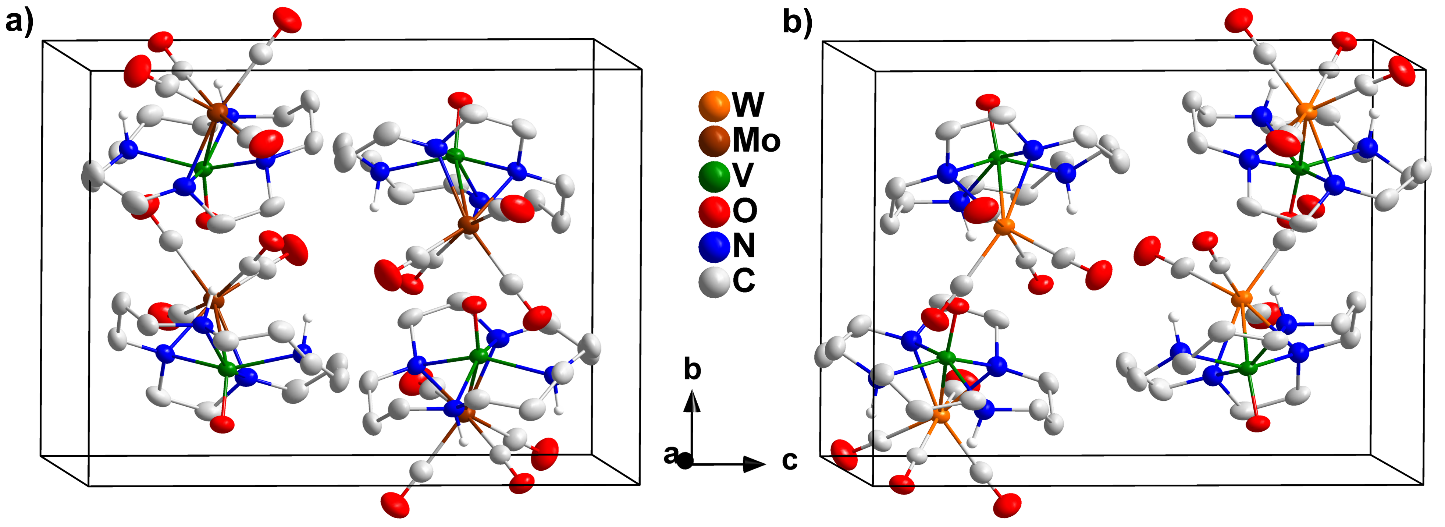
Figure S6.** Unit cells of a) VO(H_2_Cyclal)Mo(CO)_4_ (**5**), b) VO(H_2_Cyclal)W(CO)_4_ (**6**) (H atoms only partially shown for clarity).

**5. Characterization of Compounds 1-6**

To confirm the results of the single crystal structure analysis and to verify the composition and the purity of the title compounds, X-ray powder diffraction (XRD) and Fourier-transform infrared (FT-IR) spectroscopy were performed. XRD with Rietveld refinement of **3** confirms the space-group symmetry as obtained by single-crystal structure analysis (Figure S7a). Moreover, XRD confirms the purity of the title compound as no Bragg reflexes of different from **3** are observed. FT-IR spectra of **3** show *ν*(Ga‑Cl) vibrations at 415 cm^–1^,^[S6]^ vibrations of the Cp ligands (e.g. *ν*(C‑H): 3096 cm^–1^)^[S7]^ and vibrations of the THF ligand (e.g. *ν*(C-H): 3000-2800 cm^–1^, *ν*(C‑O): 1050-800 cm^–1^) (Figure S7b). FT-IR spectra of **3** are also compared to spectra of [Cp_2_MoCl_2_], GaCl_3_, and THF, which further validates the presence of the respective ligands and the origin of the vibrations for **3** (Figure S7b). FT-IR spectra of **4** show *ν*(Ga–Cl) vibrations at 413 cm^–1^,^[S6]^ vibrations of the Cp ligands (e.g. *ν*(C–H): 3098 cm^–1^, *π*(C–H): 838 cm^–1^)^[S7]^ and vibrations of the [BMIm]^+^ cation (e.g. *ν*(C–H): 3150-2700 cm^−1^; *δ*(C–H): 1560, 1163 cm^−1^) (Figure S8).^[S8]^ The absence of any O–H vibrations confirms the purity of the sample, which is especially relevant due to its air- and moisture sensitivity. FT-IR spectra of **4** are also compared to spectra of [Cp_2_MoCl_2_], GaCl_3_, and THF, which further validates the presence of the respective ligands and the origin of the vibrations for **4** (Figure S8).


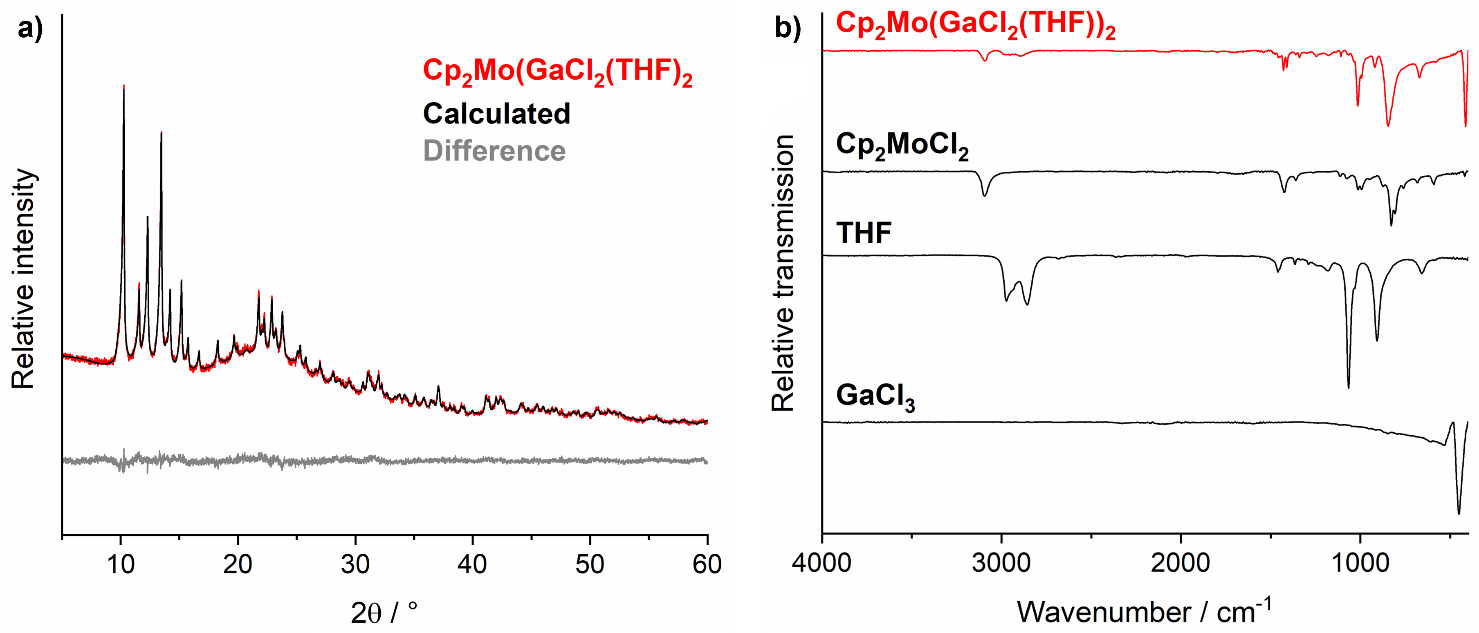


**Figure S7.** Characterization of [Cp_2_Mo(GaCl_2_(THF))_2_] (**3**): (a) XRD (data recorded at 25 °C) with Rietveld refinement (based on data from single-crystal structure analysis as structure model with data recorded at 210 K) with experimental diffractogram (red), Rietveld refinement (black), difference curve (grey); (b) FT-IR spectrum (Cp_2_MoCl_2_, THF, GaCl_3_ as references).


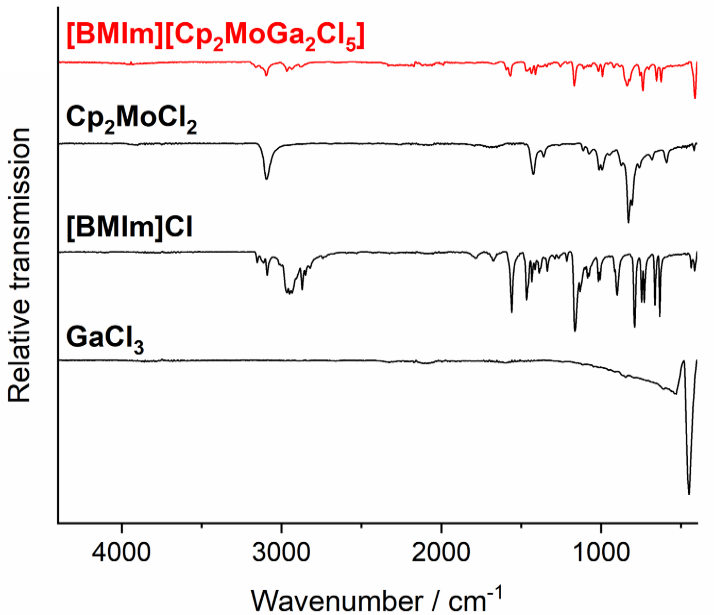


**Figure S8.** FT-IR spectrum of [BMIm][Cp_2_MoGa_2_Cl_5_] (**4**) (reference spectra of Cp_2_MoCl_2_, [BMIm]Cl, GaCl_3_).

XRD with Rietveld refinement of **6** confirms the space-group symmetry as obtained by single-crystal structure analysis (Figure S9a). Moreover, XRD confirms the purity of the title compound as no Bragg reflexes of different from **6** are observed. FT-IR spectra of **6** confirm the presence of the aza-crown ether and the W(CO)_4_ unit in the title compound (Figure S9b). The respective vibrations are well in agreement with H_4_Cyclal and W(CO)_6_ shown as references. The presence of N–H vibrations in the spectrum of **6** at 3300-3100 cm^–1^ with a significantly decreased intensity in comparison to free H_4_Cyclal confirms the partial deprotonation of the aza-crown ether in **6** (Figure S9b).


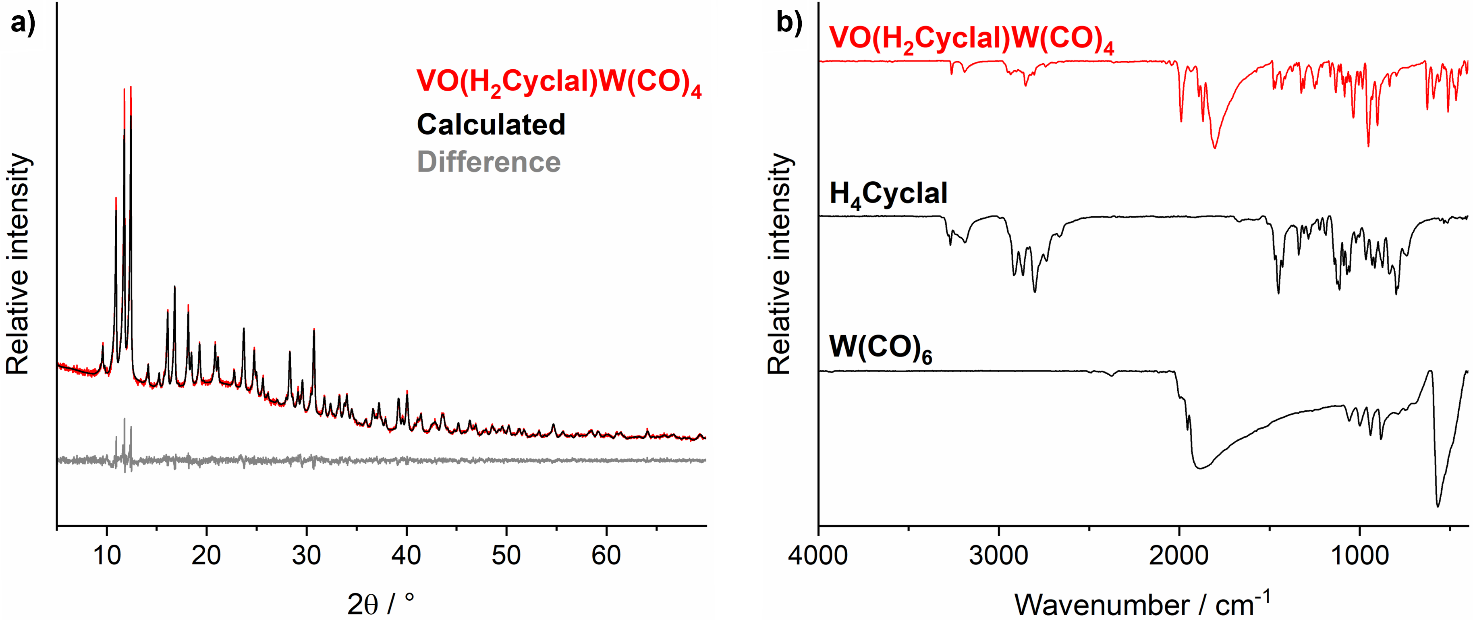


**Figure S9.** Characterization of [VO(H_2_Cyclal)W(CO)_4_] (**6**): (a) XRD (data recorded at 25 °C) with Rietveld refinement (based on data from single-crystal structure analysis as structure model; data recorded at 210 K) with experimental diffractogram (red), Rietveld refinement (black), difference curve (grey), (b) FT-IR spectrum (with H_4_Cyclal and W(CO)_6_ as references).

The stability and decomposition of [Cp_2_Mo(GaCl_2_(THF))_2_] (**3**) was studied by electrospray ionization mass spectroscopy (ESI-MS) (Figure S10-S19). Besides fragments of [Cp_2_Mo(GaCl_2_(THF))_2_] (**3**) with the ionic liquid [BMIm]Cl (i.e. [BMIm][MoGa_2_Cl_3_(Cp)_6_(THF)_3_], Figures S10,S13), interestingly, fragments of [BMIm][Tb_2_MoGaCl(Cp)(THF)_3_] (Figure S10,S11), [BMIm][TbMoCl_7_(Cp)(THF)_3_(MIm)_2_] (Figure S9,S11), [Tb_2_MoCl_3_(THF)_4_] (Figure S10,S14,S15), [Tb_2_Ga(Cp)_2_] (Figure S10,S16), [Tb_2_MoGaCl(THF)(MIm)] (Figure S10,S17), [TbMoCl_2_(Cp)(THF)_2_] (Figure S10,S18) and of GaCl_4_ (Figure S10,S19) are also visible under ESI-MS conditions. Beside the stability of the Mo–Ga bonding, this also indicates the presence of Tb–Mo–Ga and Tb–Mo species is solution as well as the interaction and formation of intermediates for the reaction of Tb(0) nanoparticles with [Cp_2_MoCl_2_]. This also suggests to perform further attempts to crystallize and identify the potential compounds. Electrospray-ionization mass spectrometry (ESI-MS) of [Cp_2_Mo(GaCl_2_(THF))_2_] (**3**) with the fragments [BMIm][MoGa_2_Cl_3_(Cp)_6_(THF)_3_], [BMIm][TbMoCl_7_(Cp)(THF)_3_(MIm)_2_]/ [BMIm][Tb_2_MoGaCl(Cp)(THF)_3_], [Tb_2_MoCl_3_(THF)_4_], [Tb_2_MoGaCl(THF)(MIm)]/ [Tb_2_Ga(Cp)_2_], [TbMoCl_2_(Cp)(THF)_2_]. High-resolution mass spectra of these fragments are displayed in Figures S11-S19.


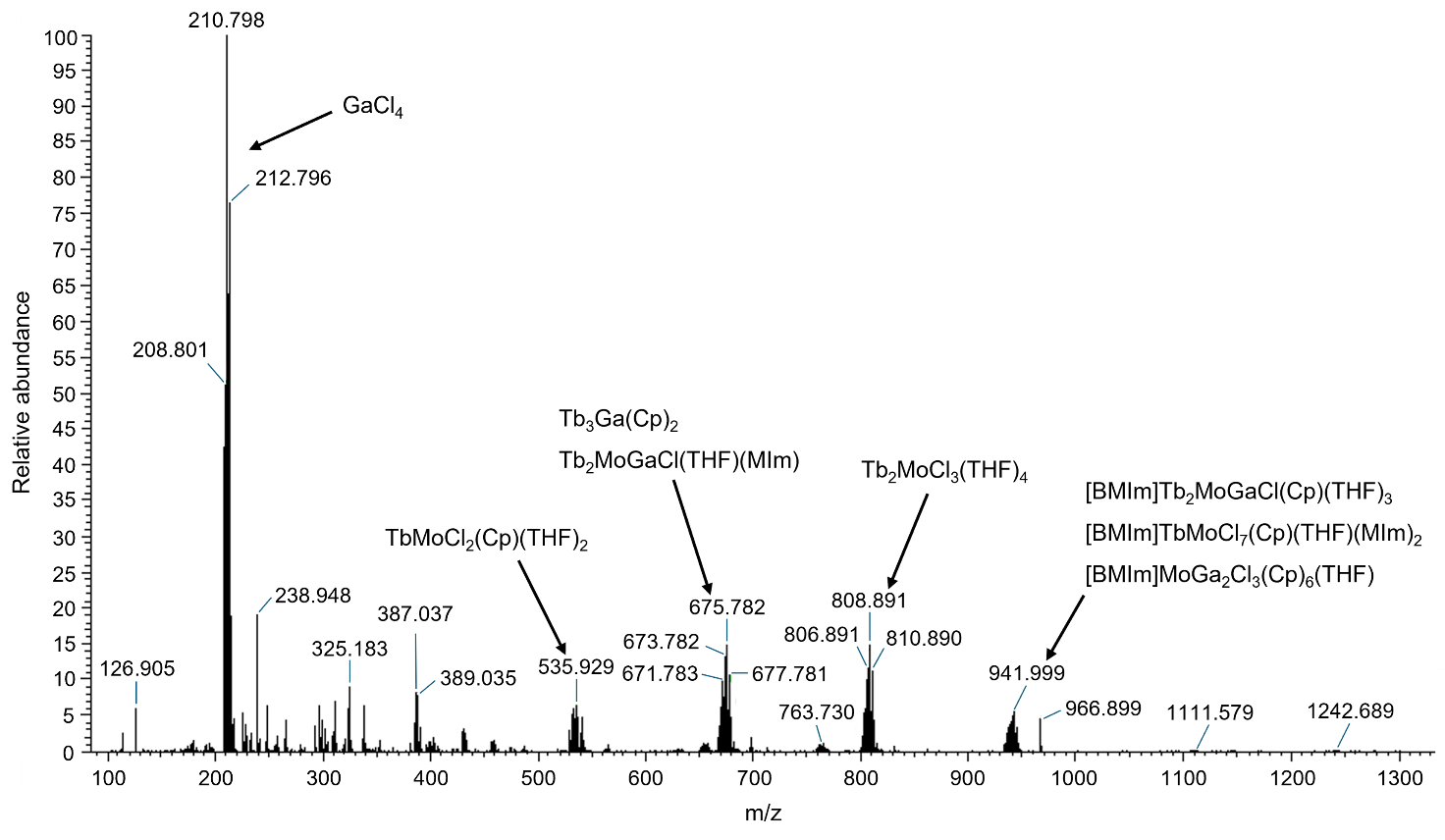


**Figure S10.** Electrospray-ionization mass spectrometry (ESI-MS) of [Cp_2_Mo(GaCl_2_(THF))_2_] (**3**) with the fragments [BMIm][MoGa_2_Cl_3_(Cp)_6_(THF)_3_], [BMIm][TbMoCl_7_(Cp)(THF)_3_(MIm)_2_]/ [BMIm][Tb_2_MoGaCl(Cp)(THF)_3_], [Tb_2_MoCl_3_(THF)_4_], [Tb_2_MoGaCl(THF)(MIm)]/ [Tb_2_Ga(Cp)_2_], [TbMoCl_2_(Cp)(THF)_2_]. High-resolution mass spectra of these fragments are displayed in Figures S11-S19.


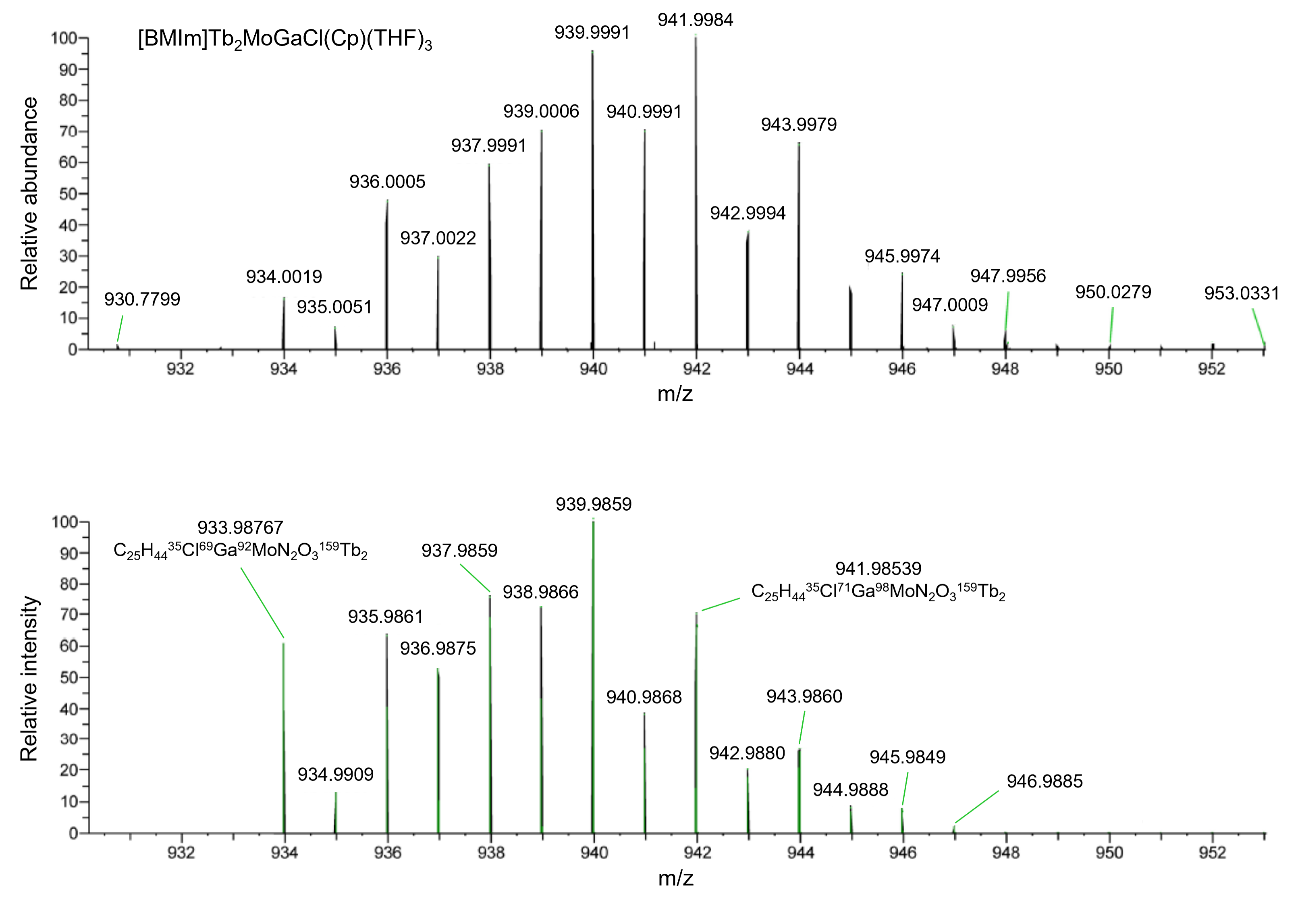


**Figure S11.** High-resolution mass spectra of the [BMIm][Tb_2_MoGaCl(Cp)(THF)_3_] fragment with the measured spectrum (top) and the simulated spectrum (bottom).


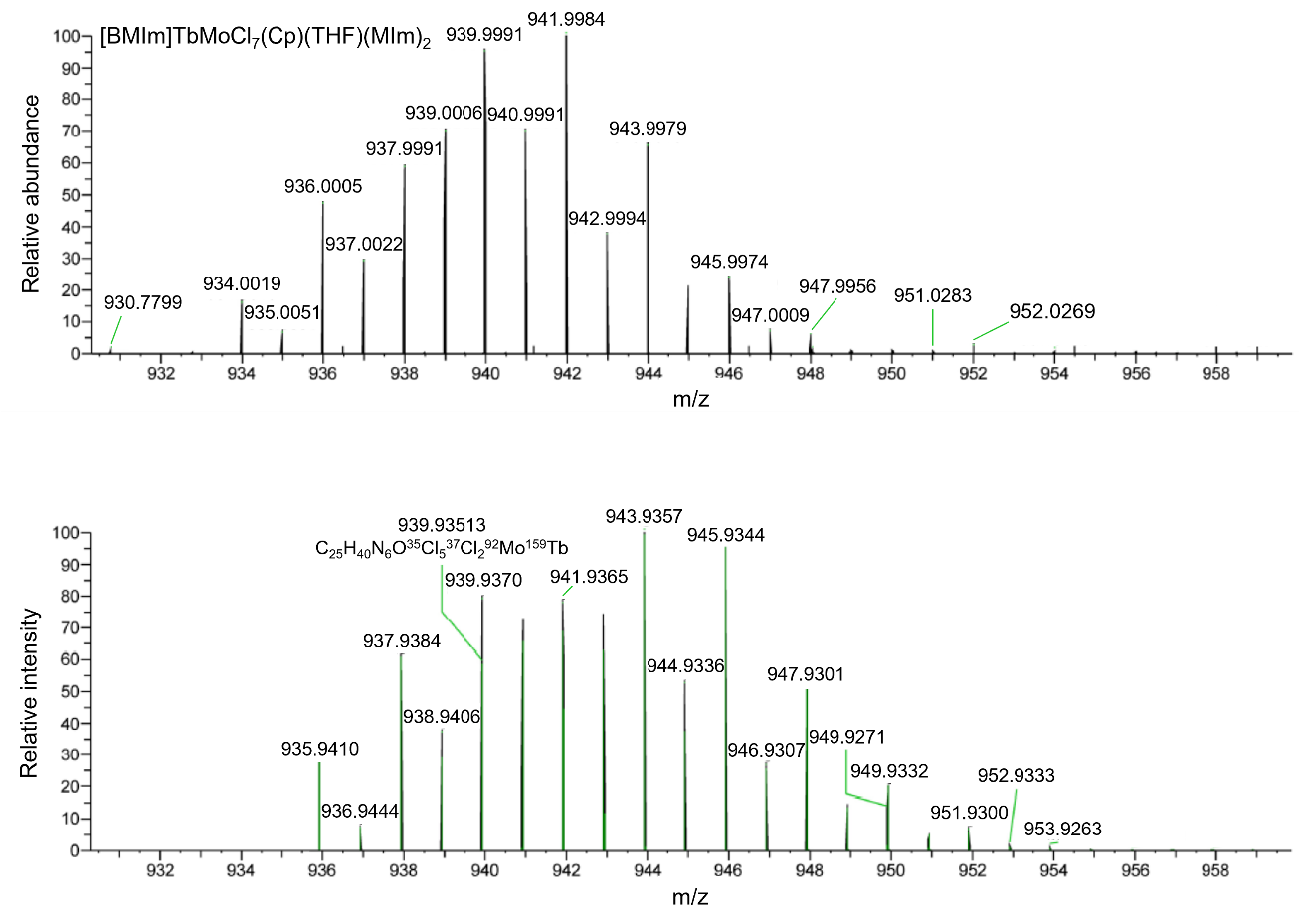


**Figure S12.** High-resolution mass spectra of the [BMIm][TbMoCl_7_(Cp)(THF)_3_(MIm)_2_] fragment with the measured spectrum (top) and the simulated spectrum (bottom).


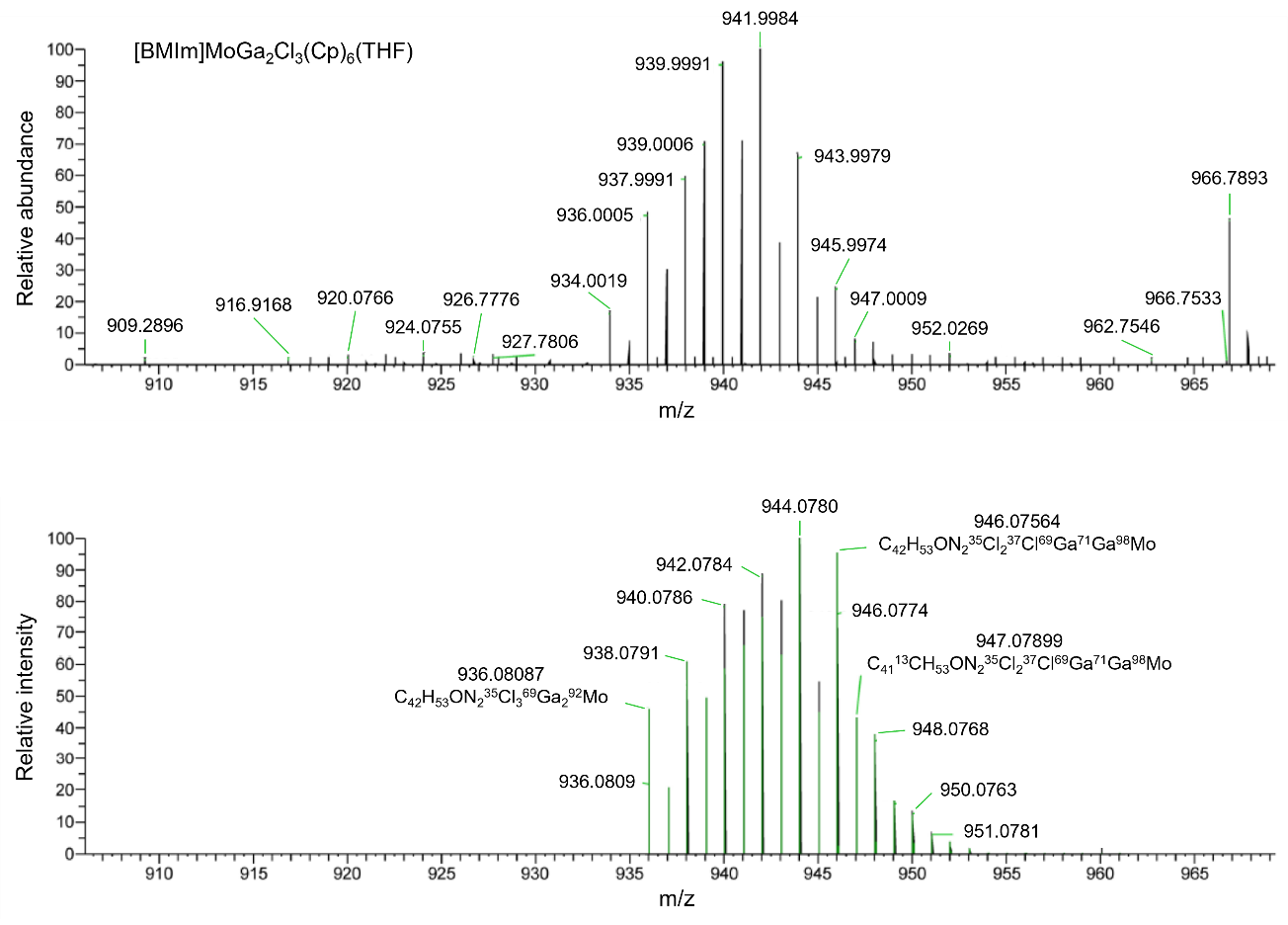


**Figure S13.** High-resolution mass spectra of the [BMIm][MoGa_2_Cl_3_(Cp)_6_(THF)_3_] fragment with the measured spectrum (top) and the simulated spectrum (bottom).


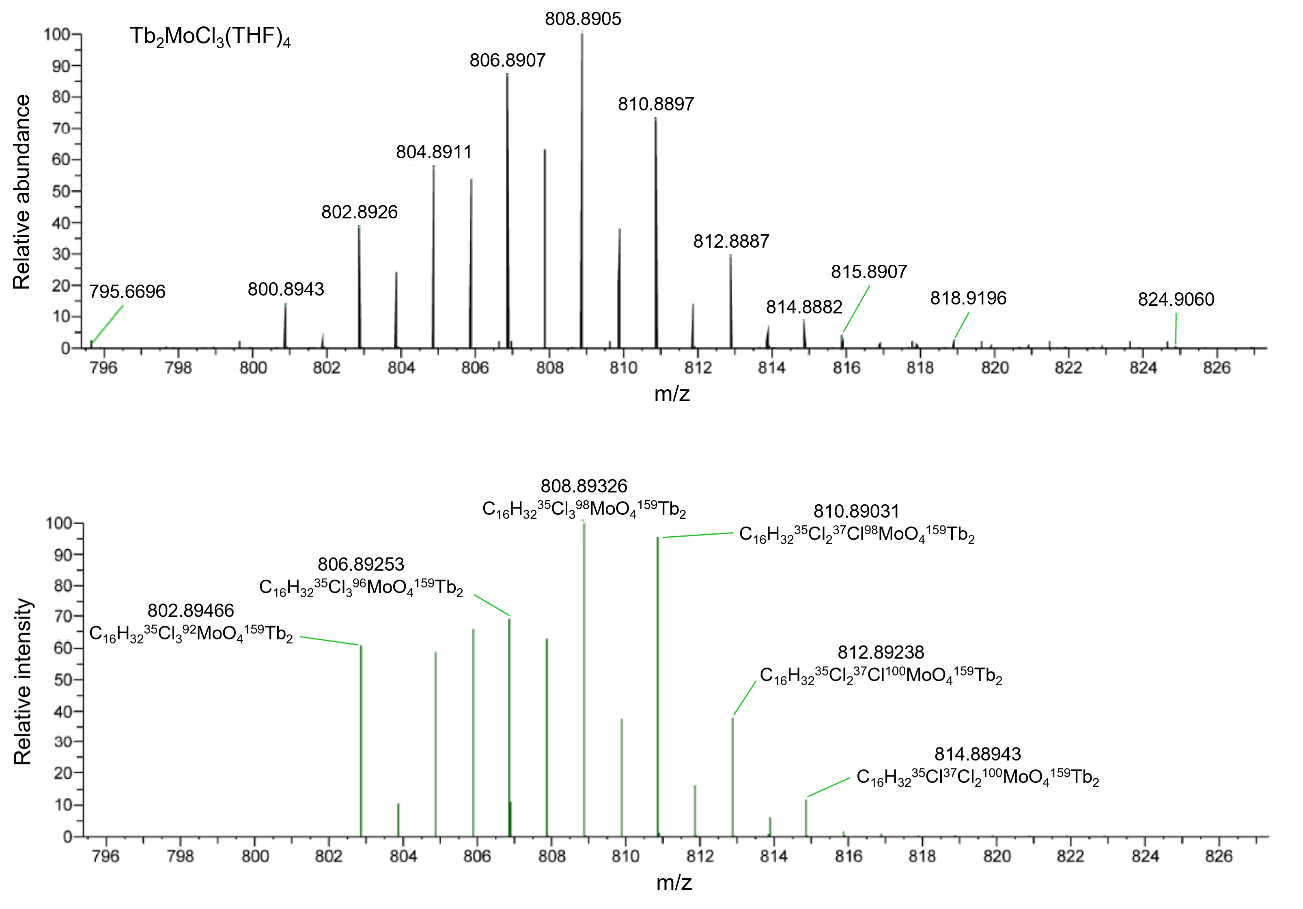


**Figure S14.** High-resolution mass spectra of the [Tb_2_MoCl_3_(THF)_4_] fragment with the measured spectrum (top) and the simulated spectrum (bottom).


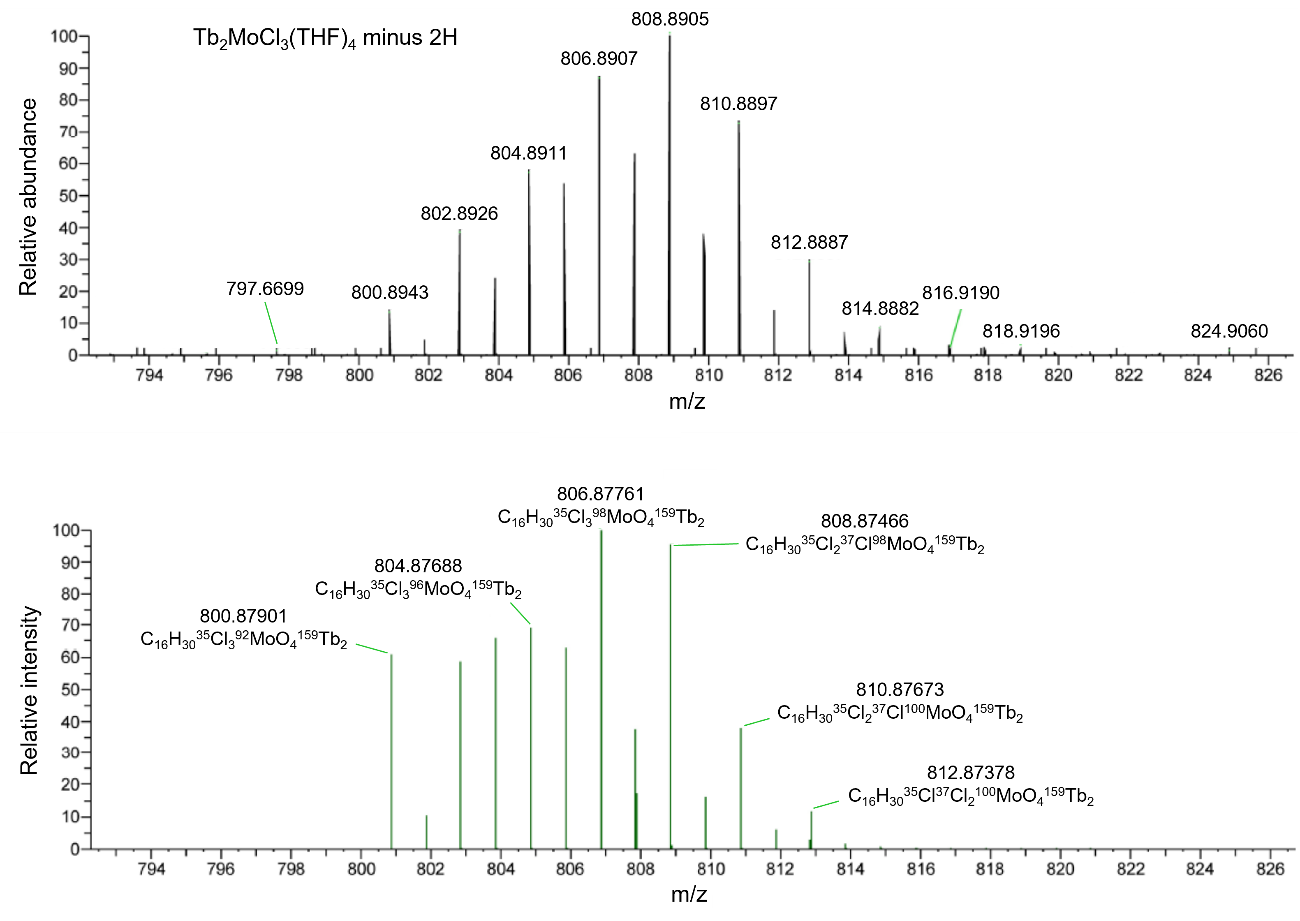


**Figure S15.** High-resolution mass spectra of the [Tb_2_MoCl_3_(THF)_4_] fragment (minus two H atoms) with the measured spectrum (top) and the simulated spectrum (bottom).


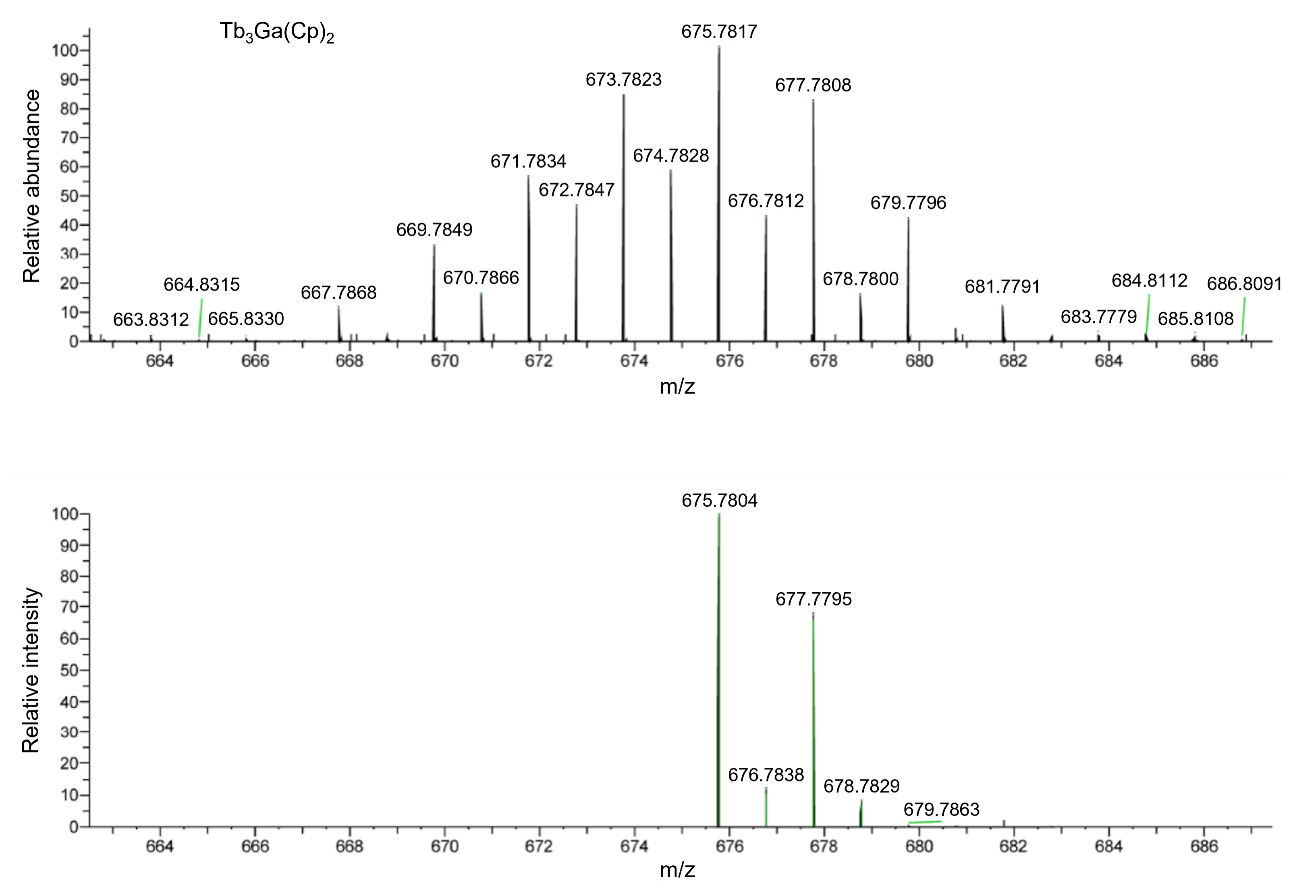


**Figure S16.** High-resolution mass spectra of the [Tb_2_Ga(Cp)_2_] fragment with the measured spectrum (top) and the simulated spectrum (bottom).


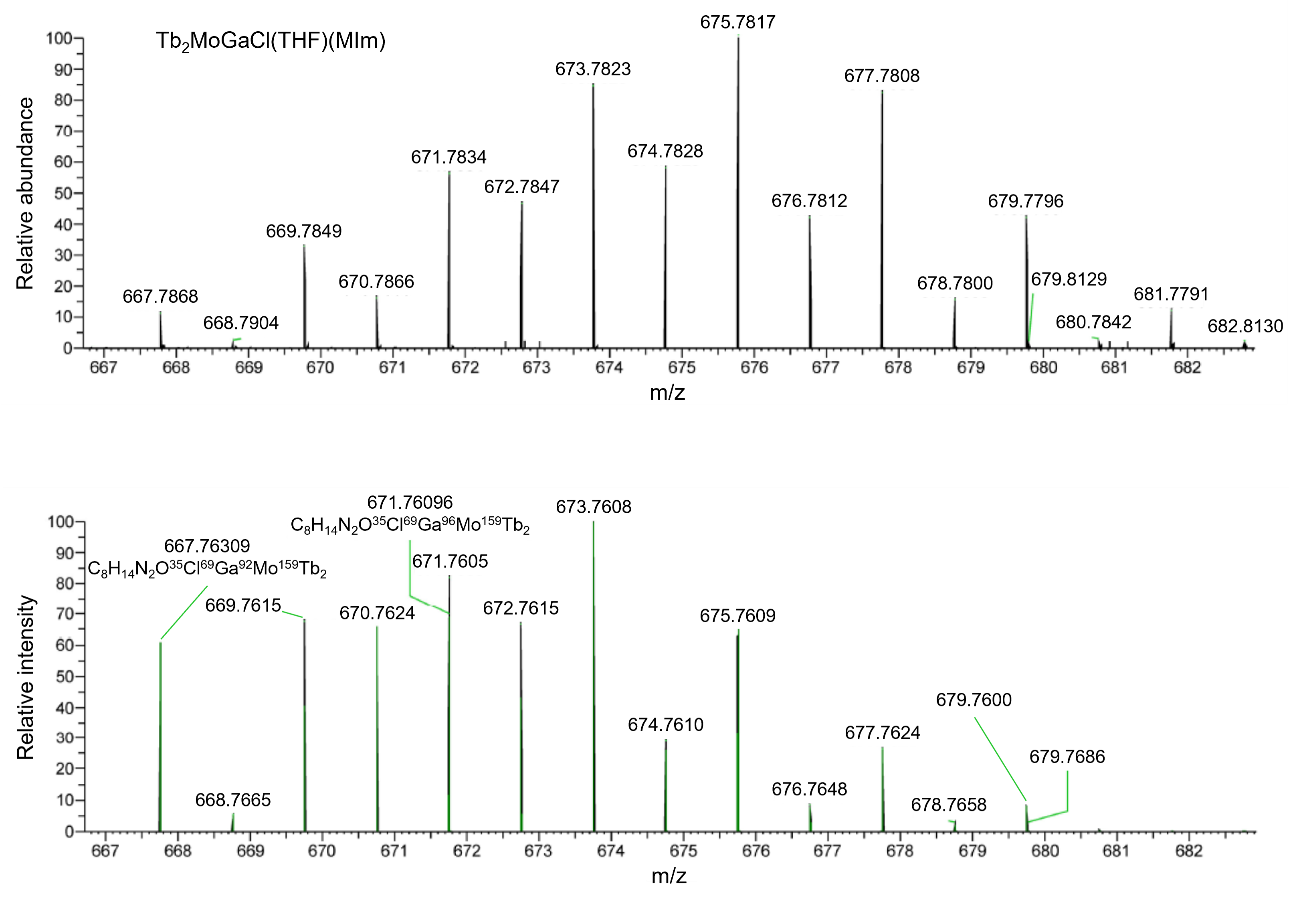


**Figure S17.** High-resolution mass spectra of the [Tb_2_MoGaCl(THF)(MIm)] fragment with the measured spectrum (top) and the simulated spectrum (bottom).


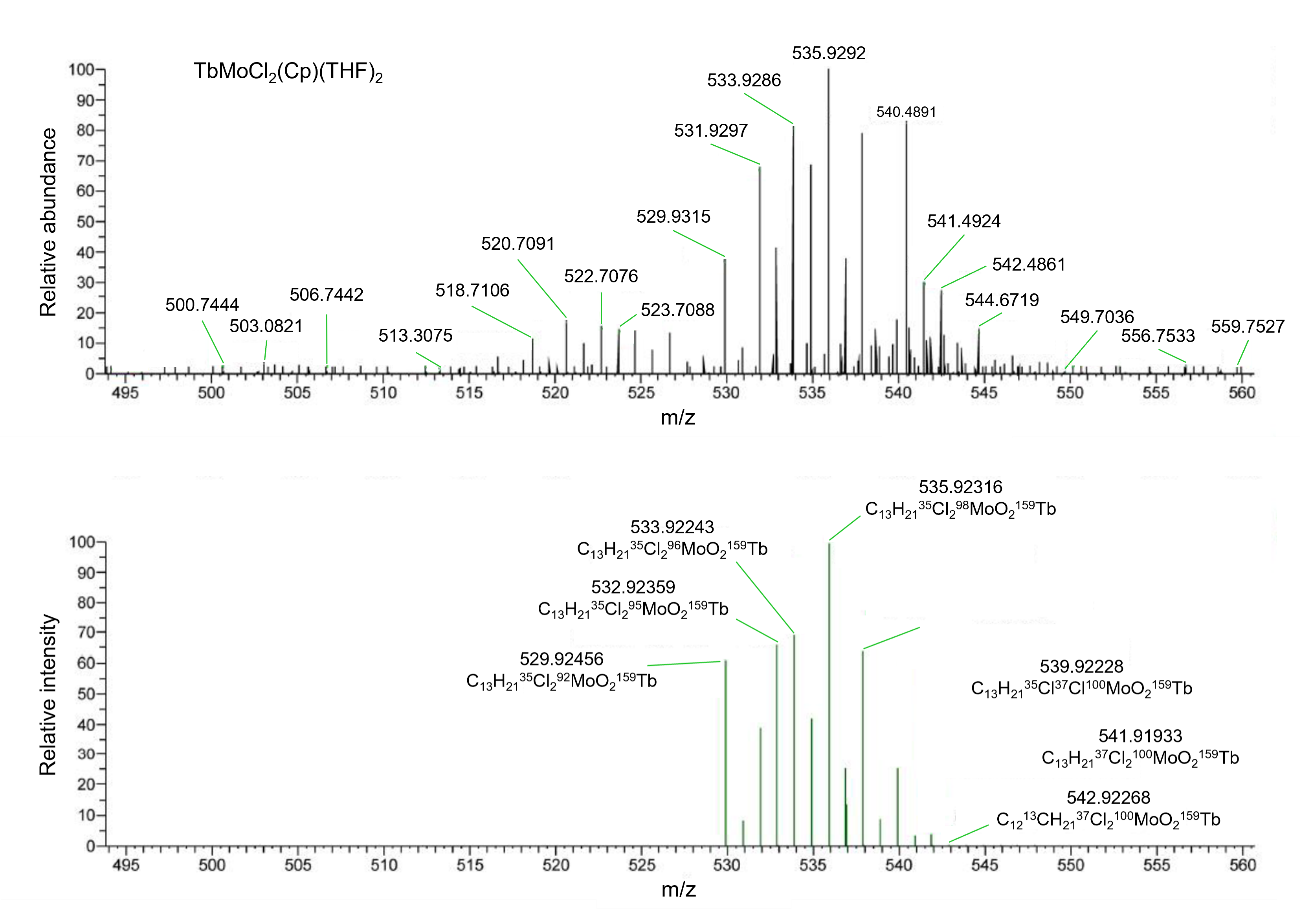


**Figure S18.** High-resolution mass spectra of the [TbMoCl_2_(Cp)(THF)_2_] fragment with the measured spectrum (top) and the simulated spectrum (bottom).


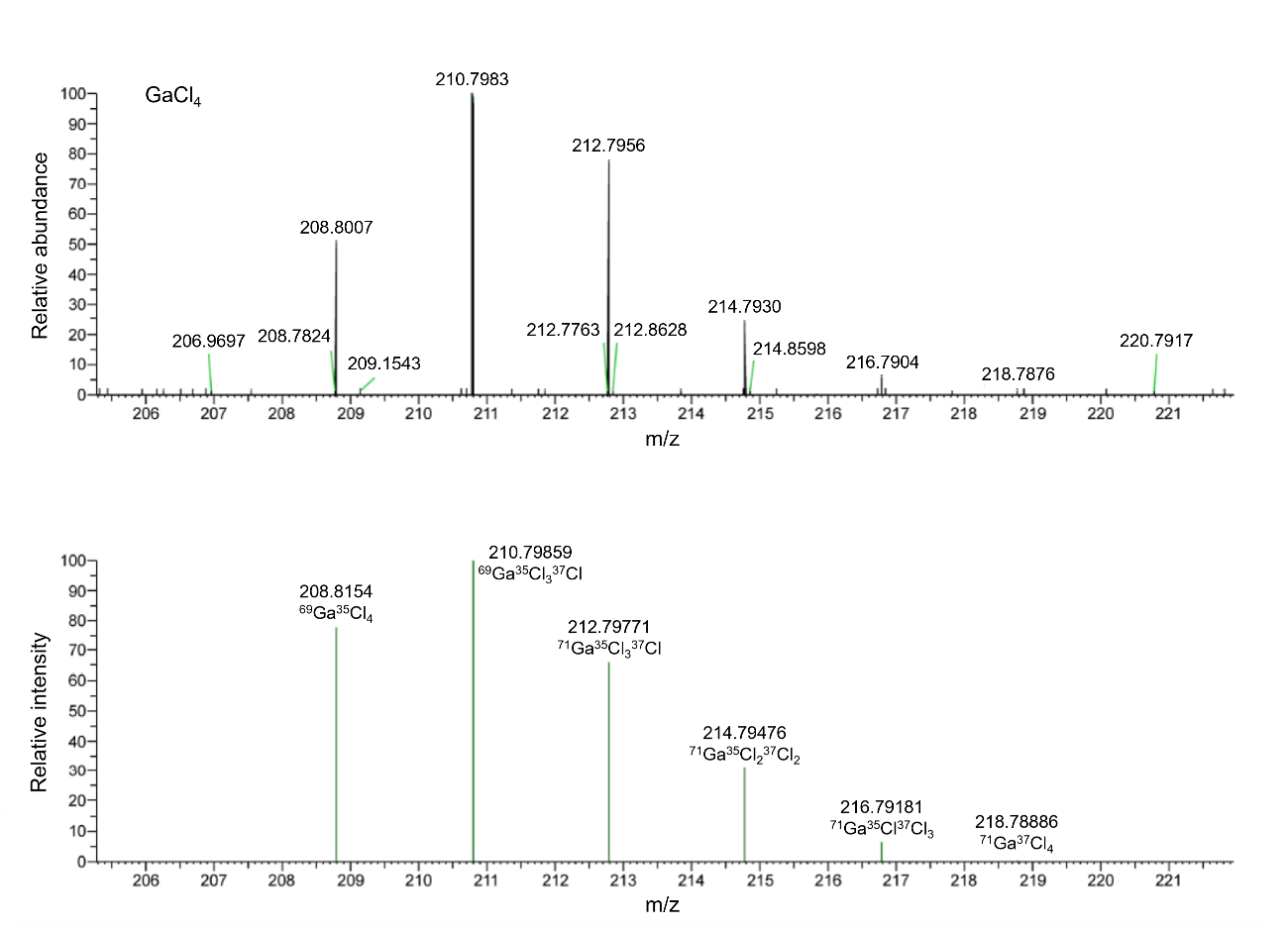


**Figure S19.** High-resolution mass spectra of the GaCl_4_ fragment with the measured spectrum (top) and the simulated spectrum (bottom).

**6. Computation**

The electronic structure and properties of the compounds **1**-**6** were investigated using density functional theory (DFT) within the resolution-of-the-identity (RI) approximation as implemented in the TURBOMOLE program package.^[S9]^ The functionals B3LYP,^[S10]^ CAM-B3LYP,^[S11]^ PBE0,^[S12]^ and TPSS^[S13]^ in combination with the def2-SVP^[S14]^ and def2-TZVP^[S15]^ basis sets for one-component calculations as well as the dhf-SVP-2c and dhf-TZVP-2c^[S16]^ basis sets for two-component calculations were used, including the corresponding relativistic effective core potentials (RECP) for the Mo and W atoms.

The equilibrium ground-state geometries were optimized using TURBOMOLE's jobex script within the unrestricted Kohn-Sham (UKS) formalism (*see main paper: Figures 4,7*). The convergence criterion of 10^–7^ *E_h_* for the change in the DFT energy and TURBOMOLE's grid size m3 were applied throughout all calculations. In case of the [Cp_2_Mo(GaCl_2_(THF))_2_] complex, different structures were investigated. Here, we obtained a structure displaying *C_2_* symmetry and an energy that is by 11.8 kJ/mol lower than the provided experimental structure with *C_1_* symmetry. The *C_2v_* point group for [Cp_2_MoGa_2_Cl_5_]^–^, [Cp_2_Mo(GaCl_3_)_2_]^–^, and [Cp_2_W(GaCl_3_)_2_]^–^, the *C_2_* point group for [Cp_2_Mo(GaCl_2_(THF))_2_] as well as the *C_1_* point group for [VO(H_2_Cyclal)Mo(CO)_4_] and [VO(H_2_Cyclal)W(CO)_4_] were used throughout all one-component calculations while the *C_1_* point group was applied for two-component calculations. However, the two-component geometry optimizations kept the symmetry of the respective complexes. TURBOMOLE's NumForce script was used to compute harmonic vibrational frequencies, which were all real, therefore confirming that the optimization resulted in a minimum on the potential energy surface. Furthermore, a natural population analysis (NPA, keyword “nbo”)^[S17]^ was carried out to determine the natural charges of the V, Ga, Mo, and W atoms (*SI: Table S3*).

Natural molecular orbitals (NMOs) were calculated at the B3LYP/dhf-TZVP-2c level of theory and were localized using the Pipek-Mezey method^[S18]^ as available within TURBOMOLE (Figures S20,S21). The highest occupied *d*-orbital was shown to be singly occupied for the complexes [Cp_2_Mo(GaCl_3_)_2_]^–^, [Cp_2_W(GaCl_3_)_2_]^–^, [VO(H_2_Cyclal)W(CO)_4_] and [VO(H_2_Cyclal)Mo(CO)_4_], but doubly occupied for [Cp_2_MoGa_2_Cl_5_]^–^ and [Cp_2_Mo(GaCl_2_(THF))_2_]. The respective NMO of the *d*-orbital of the V, Mo and W atoms (SI: Figure S20) as well as the localized orbitals with contributions (Table S4) from Mo–Ga or W–Ga were visualized using the VMD program.^[S19]^ The localization for [VO(H_2_Cyclal)W(CO)_4_] and [VO(H_2_Cyclal)Mo(CO)_4_] did not result in localized orbitals with contributions from both the V and *M* atoms (*M*: Mo, W), thus, excluding a V–*M* metal-metal bond.

**Table S3.** Interatomic distances (in pm), bond angles (in degree) and nbo charges for the V, Ga, Mo and W atoms of the various complexes. Optimized in the dhf-TZVP-2c basis set for four different functionals.

| **[Cp_2_MoGa_2_Cl_5_]^–^** | | | | |
| --- | --- | --- | --- | --- |
|  | **Mo–Ga [pm]** | **Ga–Mo–Ga [deg]** | **nbo charge Mo** | **nbo charge Ga** |
| **B3LYP** | 264.38 | 72.64 | -0.48 | 1.10 |
| **CAM-B3LYP** | 260.72 | 72.12 | -0.50 | 1.12 |
| **PBE0** | 260.48 | 71.82 | -0.57 | 1.12 |
| **TPSS** | 261.65 | 72.14 | -0.56 | 1.07 |
| **[Cp_2_Mo(GaCl_3_)_2_]^–^** | | | | |
|  | **Mo–Ga [pm]** | **Ga–Mo–Ga [deg]** | **nbo charge Mo** | **nbo charge Ga** |
| **B3LYP** | 276.88 | 105.82 | -0.03 | 1.11 |
| **CAM-B3LYP** | 272.39 | 105.09 | -0.03 | 1.14 |
| **PBE0** | 272.55 | 111.08 | -0.11 | 1.14 |
| **TPSS** | 273.85 | 112.64 | -0.12 | 1.09 |
| **[Cp_2_Mo(GaCl_2_(THF))_2_]** | | | | |
|  | **Mo–Ga [pm]** | **Ga–Mo–Ga [deg]** | **nbo charge Mo** | **nbo charge Ga** |
| **B3LYP** | 260.50 | 80.08 | -0.52 | 1.16 |
| **CAM-B3LYP** | 257.71 | 79.08 | -0.54 | 1.19 |
| **PBE0** | 257.14 | 78.47 | -0.61 | 1.19 |
| **TPSS** | 258.43 | 79.13 | -0.59 | 1.14 |
| **[Cp_2_W(GaCl_3_)_2_]^–^** | | | | |
|  | **W–Ga [pm]** | **Ga–W–Ga [deg]** | **nbo charge W** | **nbo charge Ga** |
| **B3LYP** | 275.44 | 102.95 | 0.31 | 1.08 |
| **CAM-B3LYP** | 271.35 | 101.74 | 0.33 | 1.09 |
| **PBE0** | 271.76 | 108.32 | 0.25 | 1.10 |
| **TPSS** | 272.85 | 110.34 | 0.24 | 1.06 |
| **[VO(H_2_Cyclal)Mo(CO)_4_]** | | | | |
|  | **V–Mo [pm]** |  | **nbo charge Mo** | **nbo charge V** |
| **B3LYP** | 298.35 |  | -0.60 | 1.48 |
| **CAM-B3LYP** | 294.2 |  | -0.64 | 1.38 |
| **PBE0** | 292.05 |  | -0.65 | 1.47 |
| **TPSS** | 293.32 |  | -0.58 | 1.34 |
| **[VO(H_2_Cyclal)W(CO)_4_]** | | | | |
|  | **V–W [pm]** |  | **nbo charge W** | **nbo charge V** |
| **B3LYP** | 298.03 |  | -0.32 | 1.47 |
| **CAM-B3LYP** | 294.22 |  | -0.37 | 1.51 |
| **PBE0** | 292.16 |  | -0.38 | 1.46 |
| **TPSS** | 293.71 |  | -0.29 | 1.33 |

| **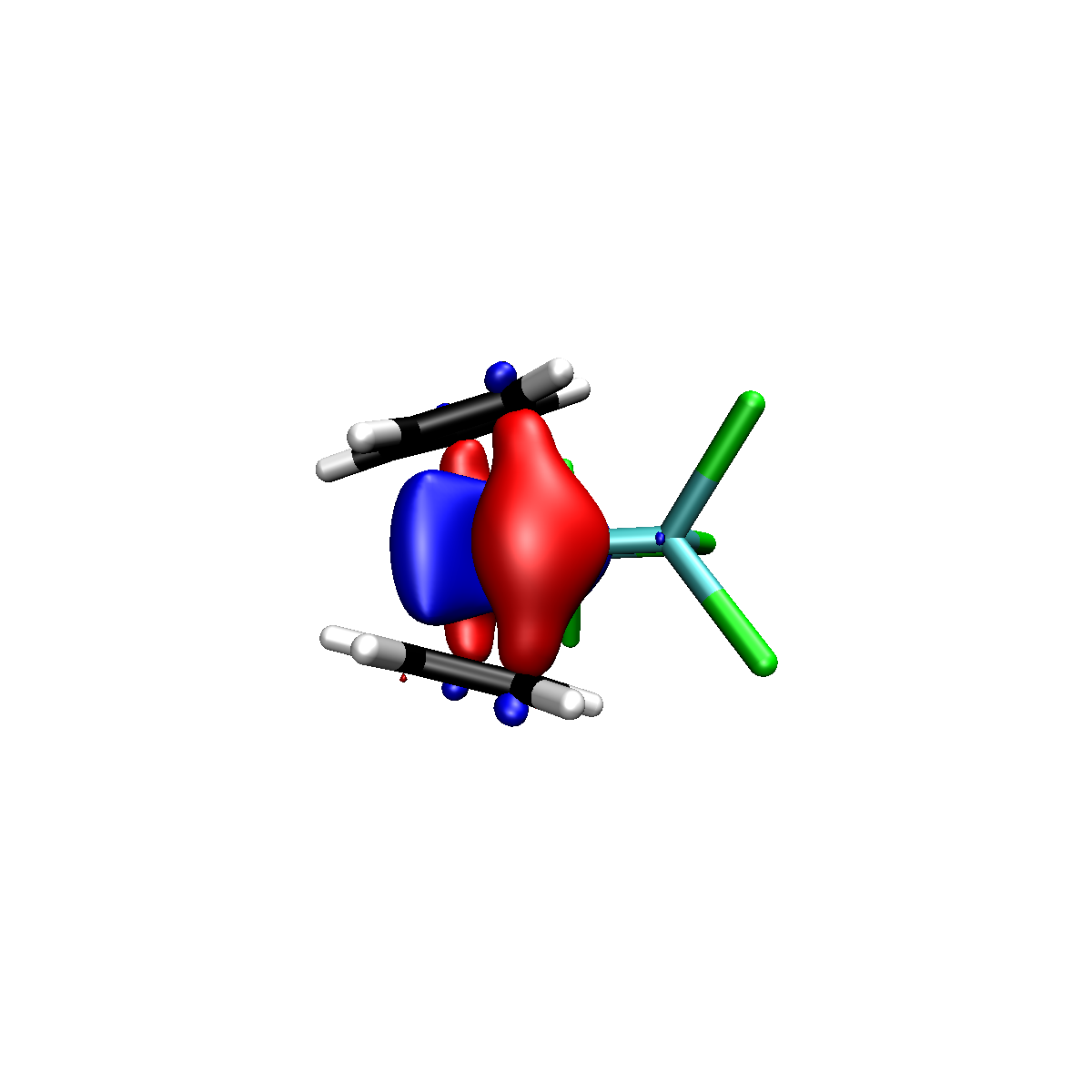**a) | **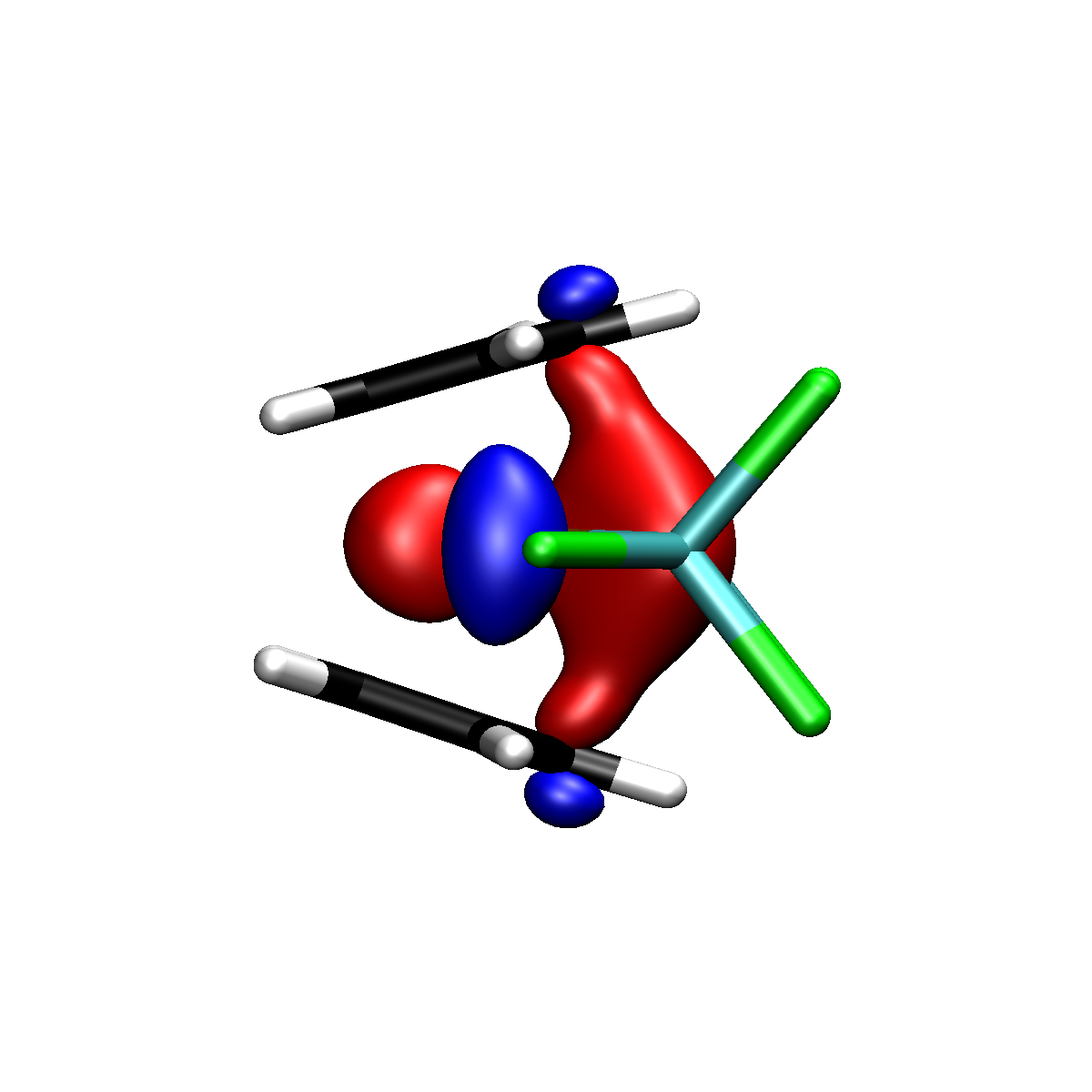**b) |
| --- | --- |
| **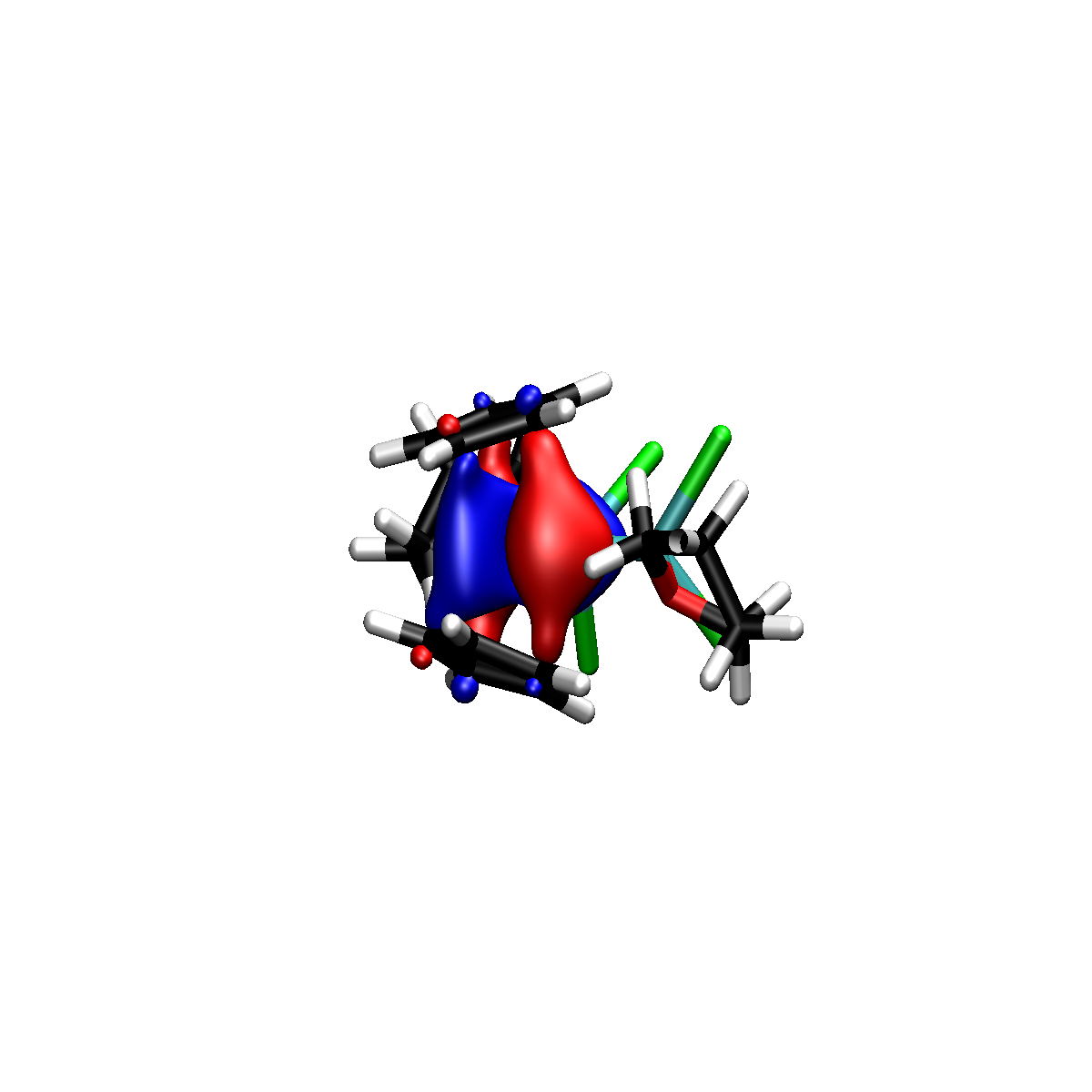**c) | **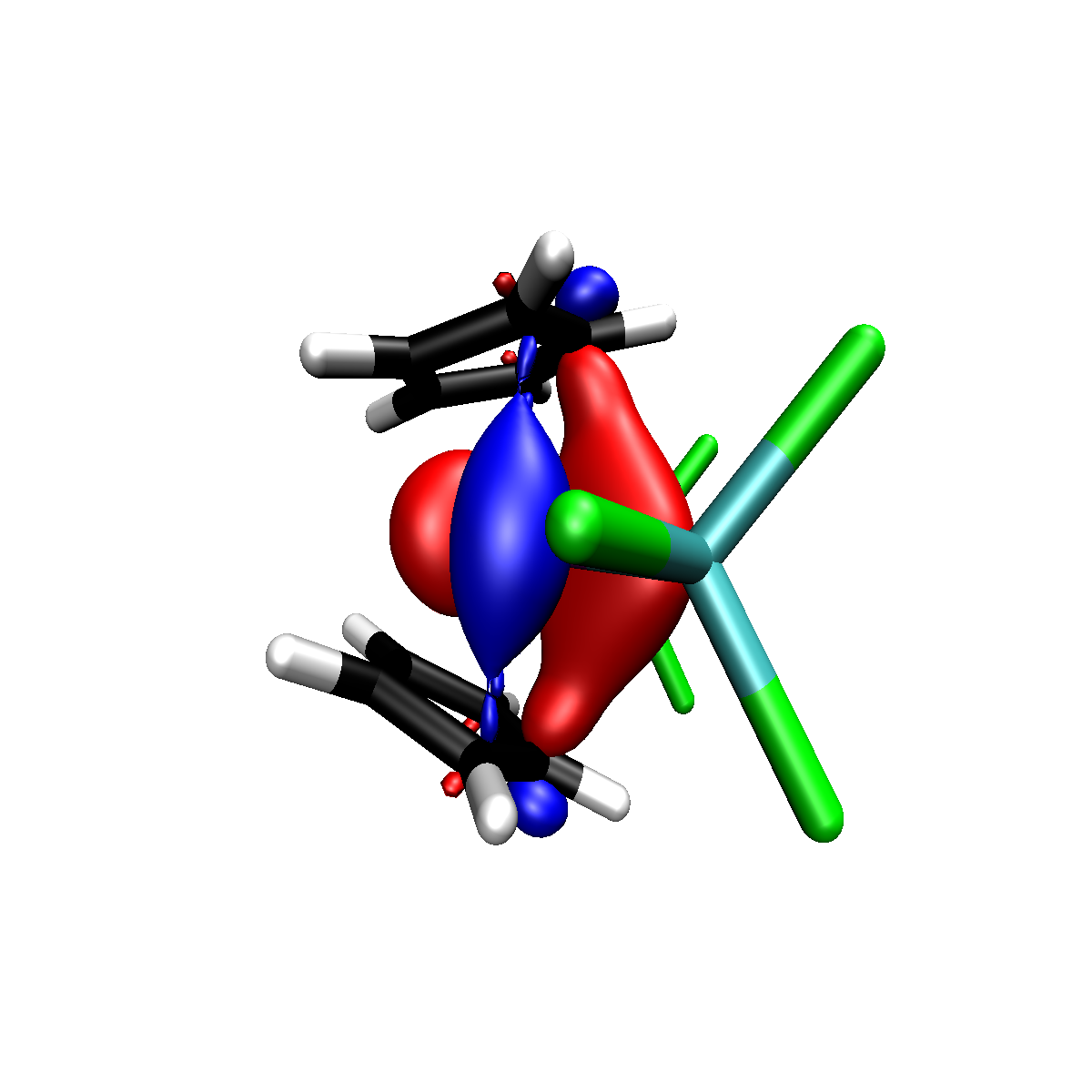**d) |
| 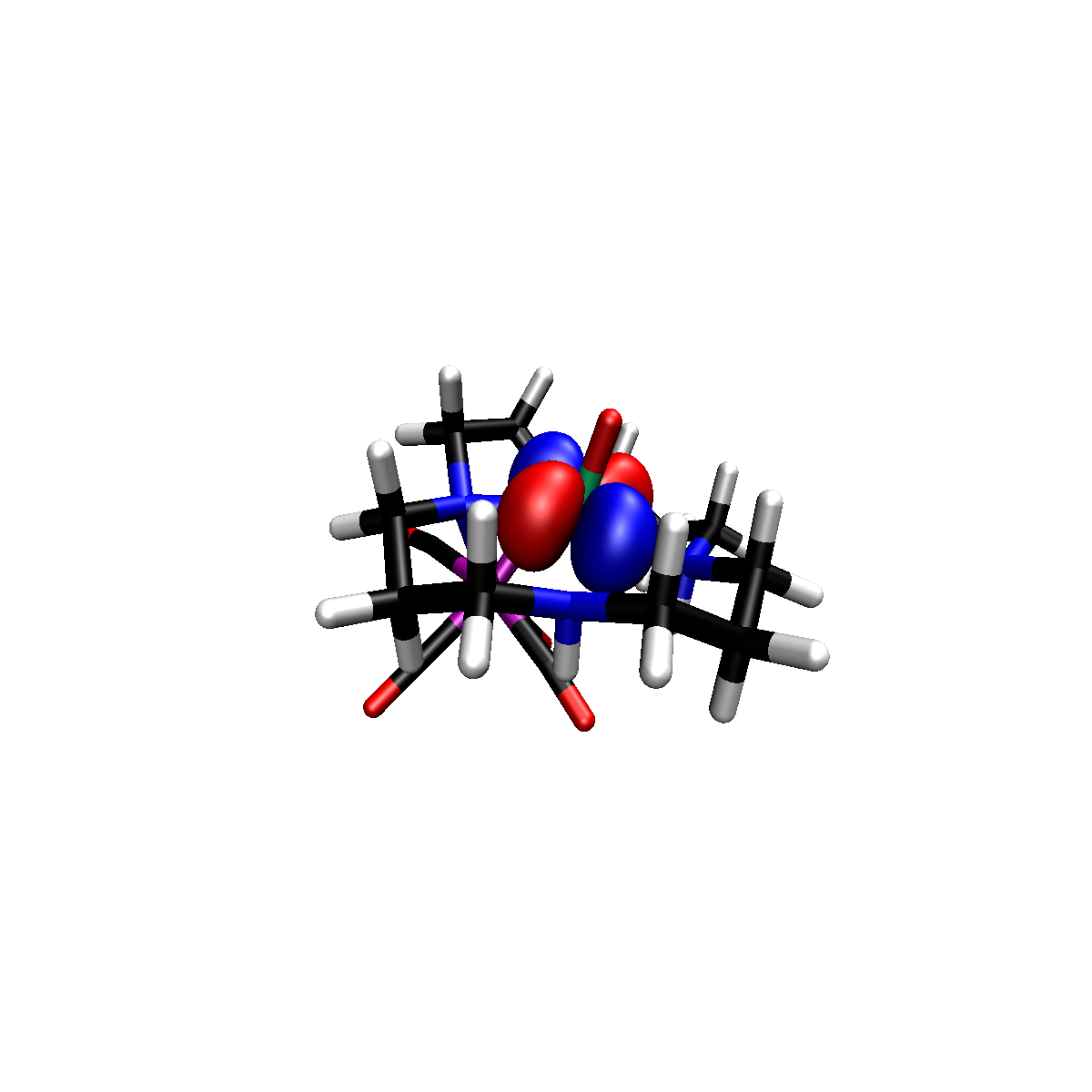e) | f)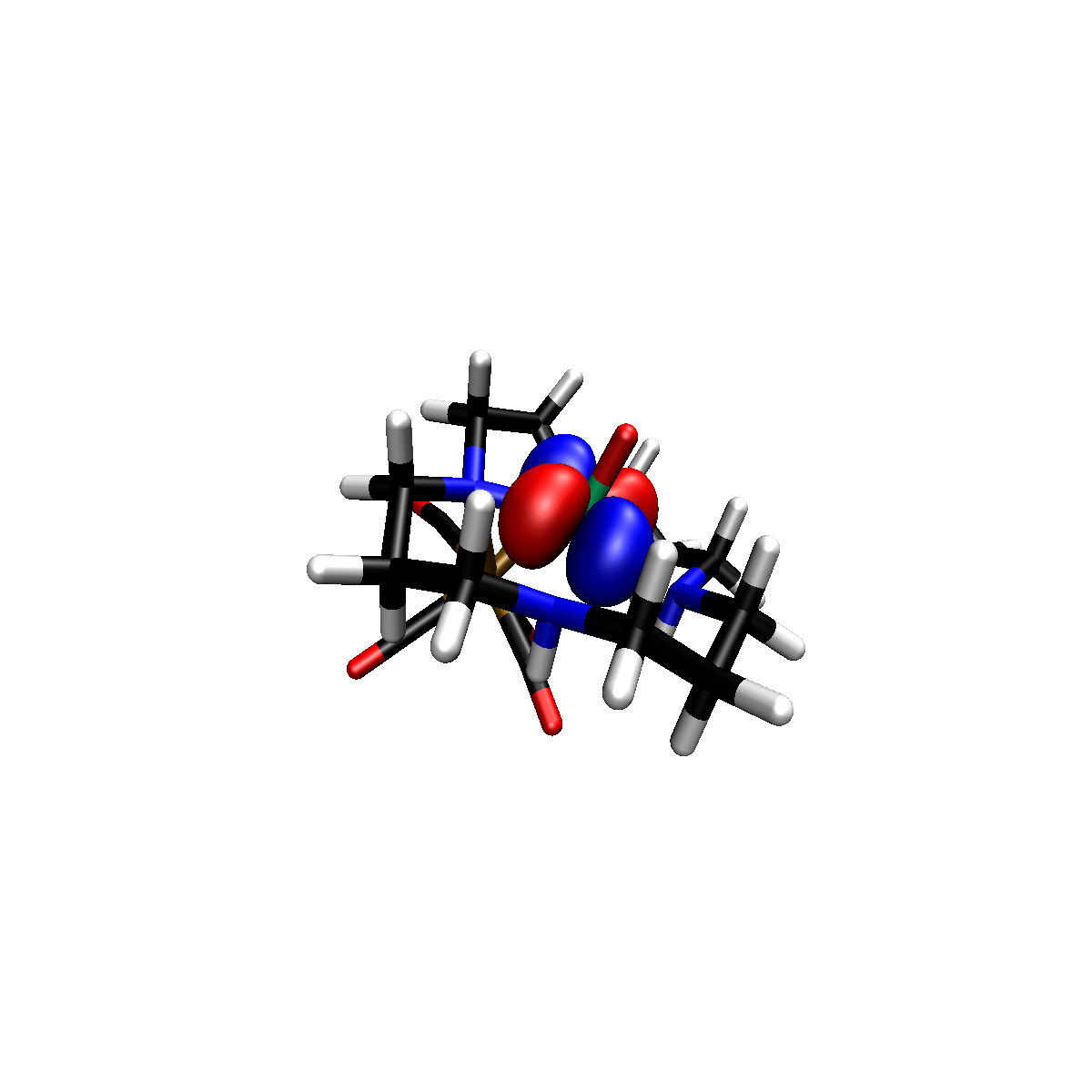 |

**Figure S20.** Natural molecular orbitals representing the highest occupied *d*-orbital located at the Mo (a-c), W (d), and V (e,f) atoms. Shown for an isovalue of ±0.04 a_0_^–3/2^ at the B3LYP/dhf-TZVP-2c level. a) [Cp_2_MoGa_2_Cl_5_]^–^ closed-shell complex with doubly occupied *d*-orbital; b) [Cp_2_Mo(GaCl_3_)_2_]^–^ open-shell complex with singly occupied *d*-orbital; c) [Cp_2_Mo(GaCl_2_(THF))_2_] closed-shell complex with doubly occupied d-orbital; d) [Cp_2_W(GaCl_3_)_2_]^-^ open-shell complex with singly occupied d-orbital; e) [VO(H_2_Cyclal)W(CO)_4_] open-shell complex with singly occupied *d*-orbital; f) [VO(H_2_Cyclal)Mo(CO)_4_] open-shell complex with singly occupied *d-*orbital.

| **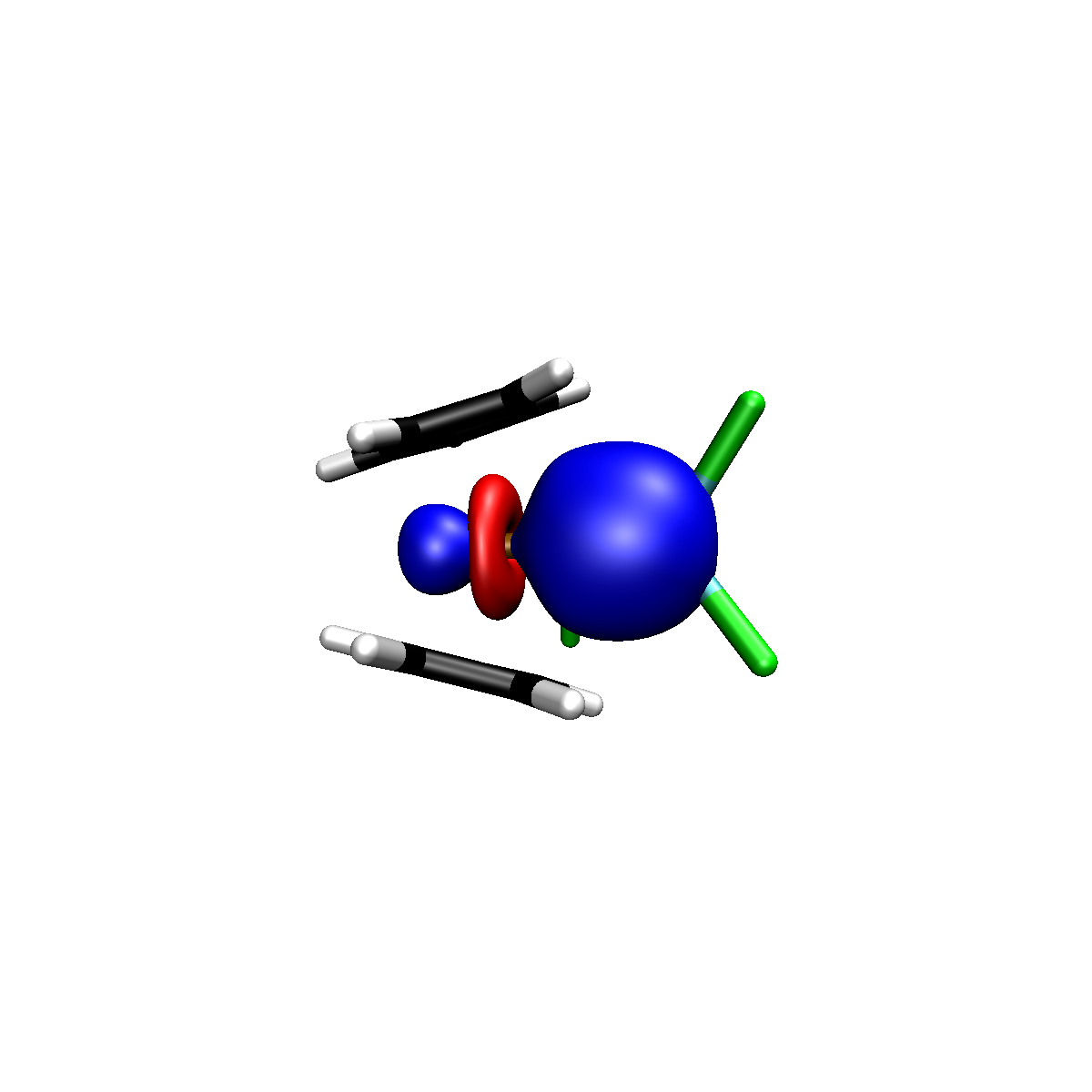**a) | **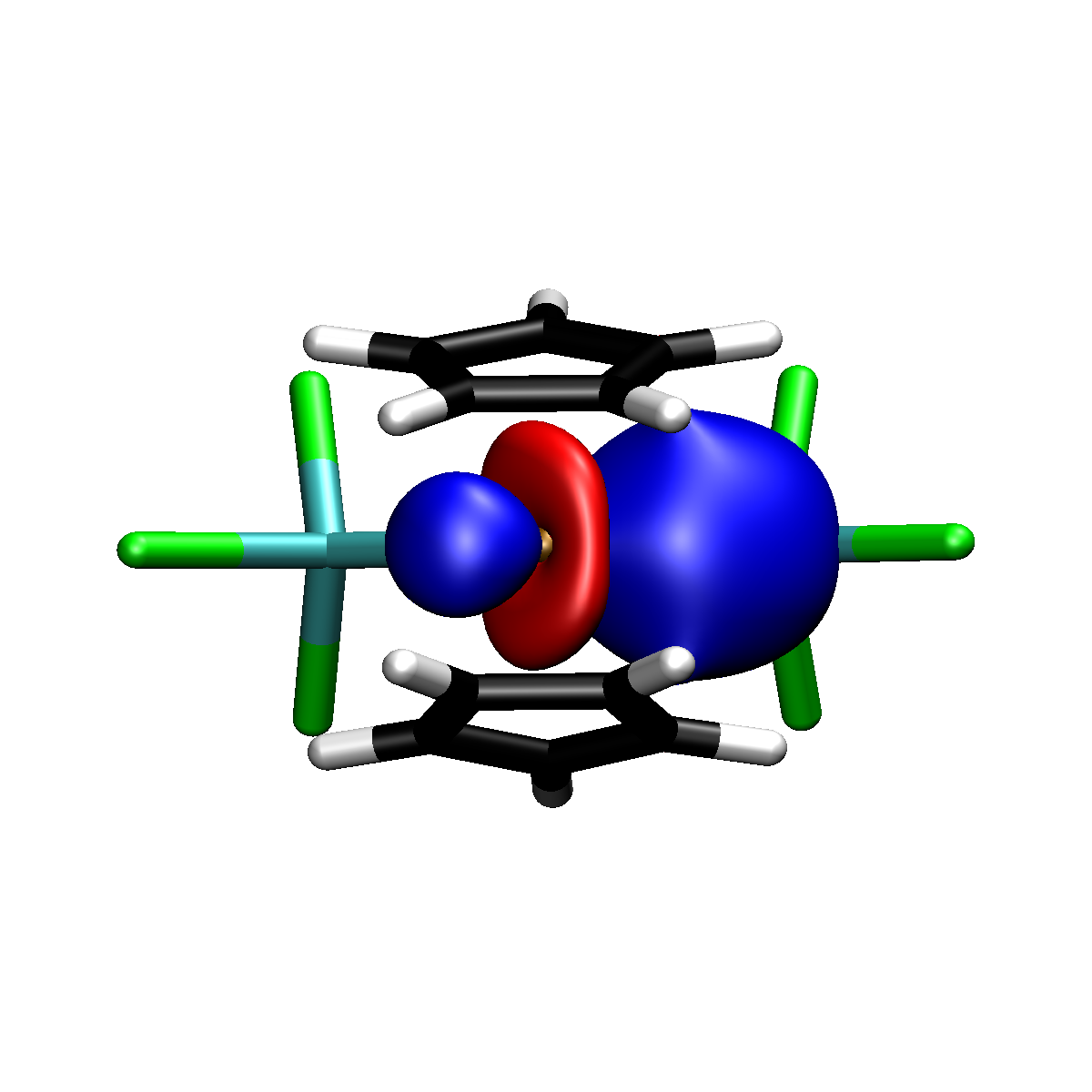**b) |
| --- | --- |
| c)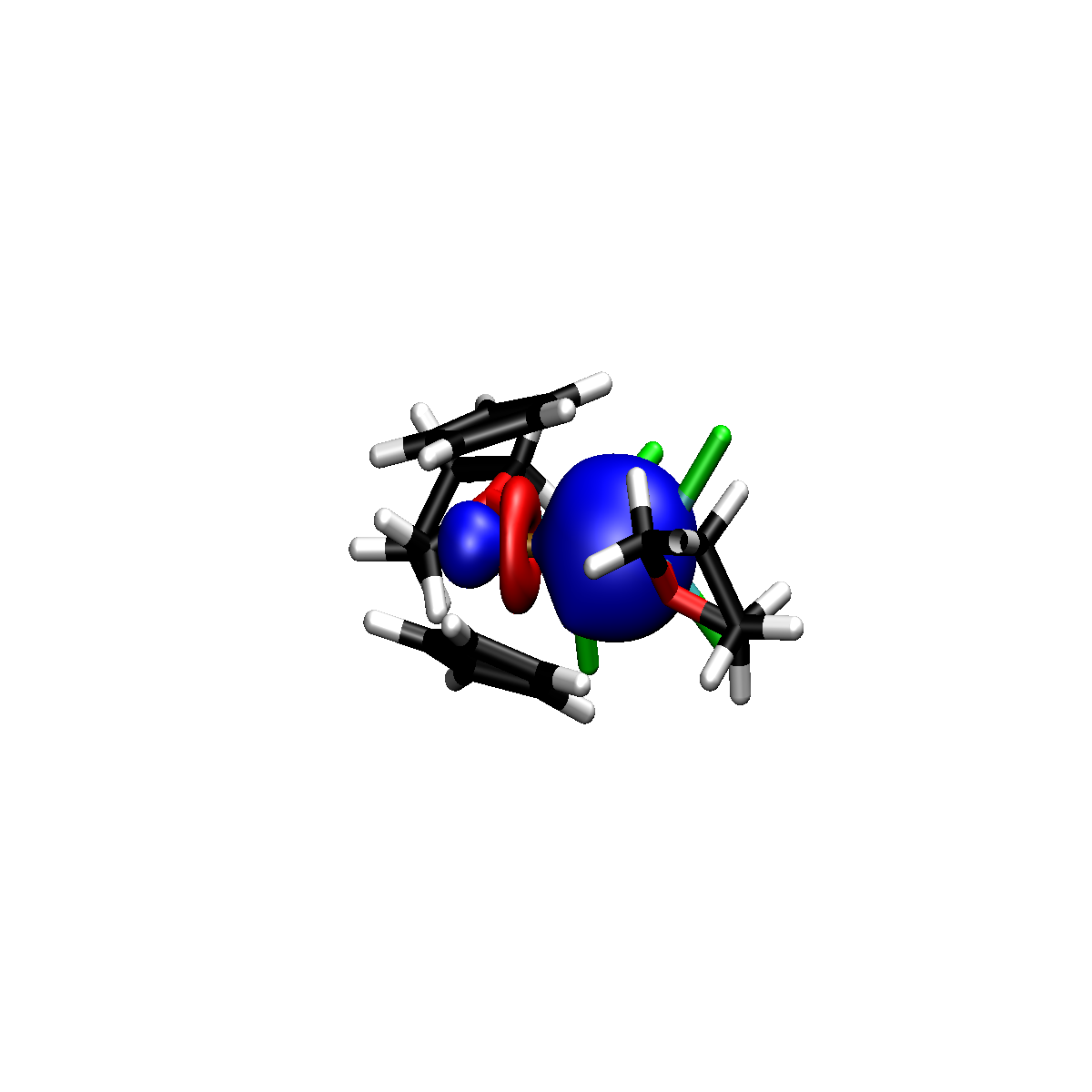 | 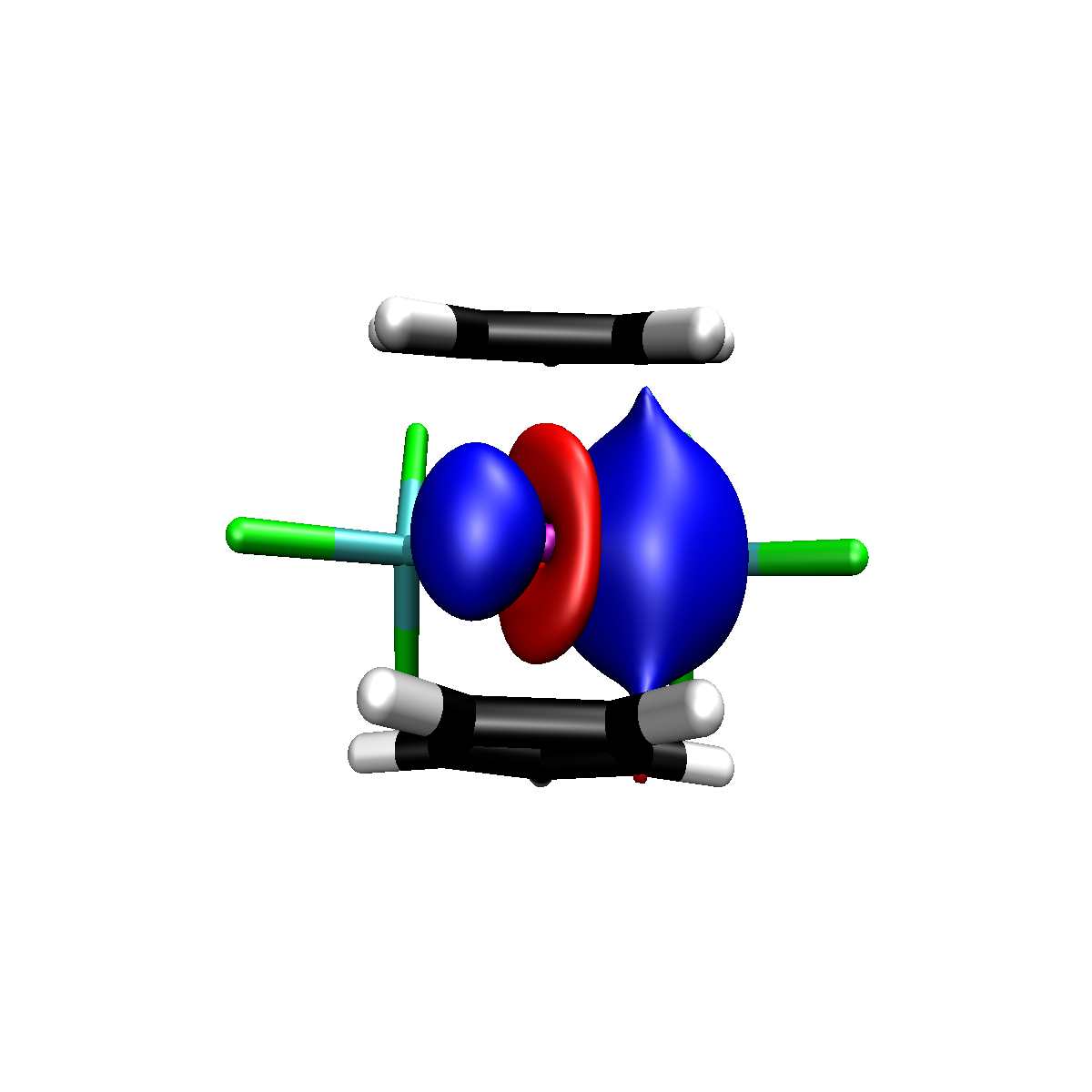d) |

**Figure S21.** Localized molecular orbitals obtained by applying a Pipek localization to B3LYP/dhf-TZVP-2c natural molecular orbitals, shown for an isovalue of ±0.04 a_0_^–3/2^: a-c) Compounds with Mo–Ga bonding; d) Compound with W–Ga bonding.

**Table S4.** Ratio of Mulliken contributions (in percent) resulting from a Pipek-Mezey localization of B3LYP/dhf-TZVP-2c natural molecular orbitals for the Mo-Ga and W-Ga bonds respectively.

|  | **[Cp_2_MoGa_2_Cl_5_]^–^ Mo : Ga** | **[Cp_2_Mo(GaCl_3_)_2_]^–^**  **Mo : Ga** | **[Cp_2_Mo(GaCl_2_(THF))_2_]**  **Mo : Ga** | **[Cp_2_W(GaCl_3_)_2_]^–^**  **W : Ga** |
| --- | --- | --- | --- | --- |
| **B3LYP** | 57 : 43 | 62 : 38 | 43 : 57 | 62 : 38 |
| **CAM-B3LYP** | 59 : 41 | 64 : 36 | 46 : 54 | 65 : 35 |
| **PBE0** | 60 : 40 | 64 : 36 | 46 : 54 | 65 : 35 |
| **TPSS** | 57 : 43 | 61 : 39 | 44 : 56 | 62 : 38 |

UV-Vis spectra were computed at the (one-component) time-dependent (TD)-DFT level in the def2-TZVP basis using the hybrid functionals B3LYP and PBE0 as well as the range-separated hybrid functional CAM-B3LYP (the non-hybrid functional TPSS is not suited for computing absorption spectra). The color prediction tool (cpt) of TURBOMOLE was used to predict the color of the compounds [VO(H_2_Cyclal)Mo(CO)_4_] and [VO(H_2_Cyclal)W(CO)_4_]. The simulated spectra are shown in Figure S22. The excitation responsible for the green color is from a metal 4*d* (5*d*) Mo (W) orbital to CO π* orbitals (Figure S22). The corresponding wavelength is given in Table S5, together with the predicted RGB color code.

**Table S5.** Wavelength (in nm) and oscillator strength (in the length representation) of the 4*d* (5*d*) to π* excitation at about 400 nm. The corresponding natural transition orbitals are displayed in Figure S22. The RGB color code as calculated from the full spectrum by TURBOMOLE’s color prediction tool is also given.

|  |  | ***λ* (nm)** | ***f*** | **R** | **G** | **B** |
| --- | --- | --- | --- | --- | --- | --- |
| **[VO(H_2_Cyclal)Mo(CO)_4_]** | **B3LYP** | 420 | 0.0070 | 199 | 255 | 9 |
|  | **CAM-B3LYP** | 400 | 0.0079 | 179 | 255 | 22 |
|  | **PBE0** | 394 | 0.0073 | 181 | 255 | 33 |
| **[VO(H_2_Cyclal)W(CO)_4_]** | **B3LYP** | 443 | 0.0058 | 218 | 255 | 16 |
|  | **CAM-B3LYP** | 423 | 0.0075 | 200 | 255 | 4 |
|  | **PBE0** | 416 | 0.0072 | 197 | 255 | 12 |

a) b)


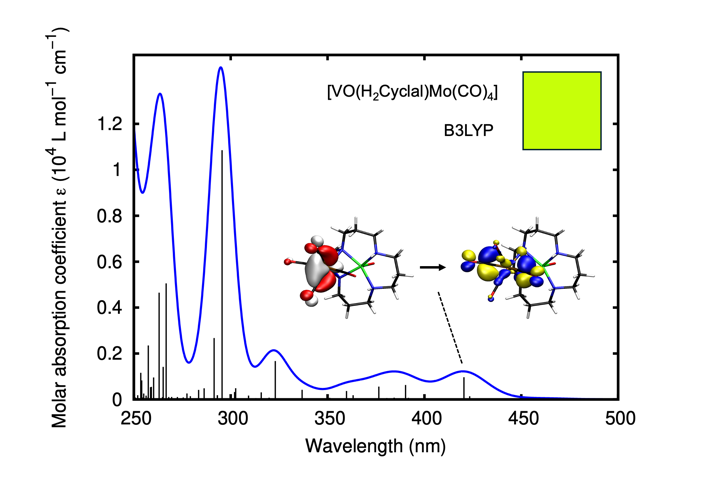

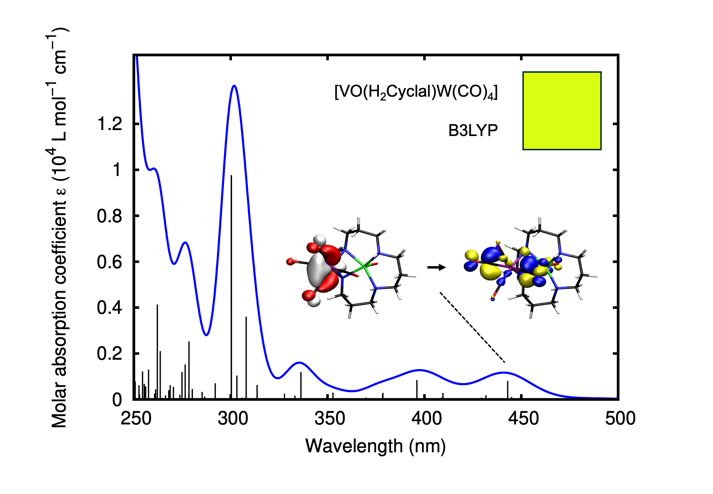


c) d)


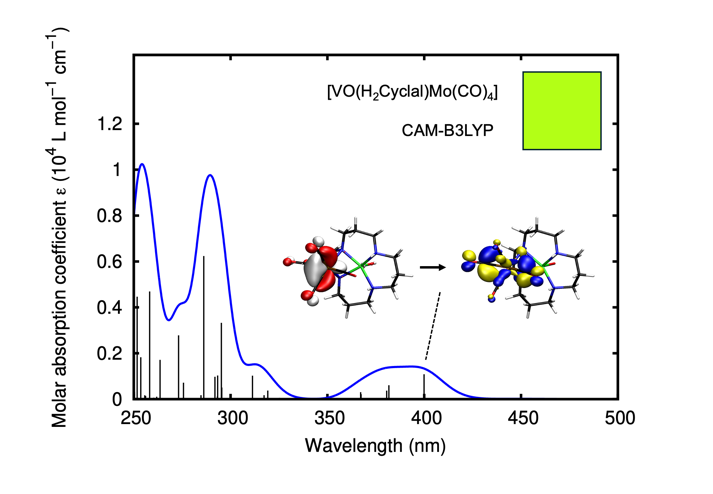

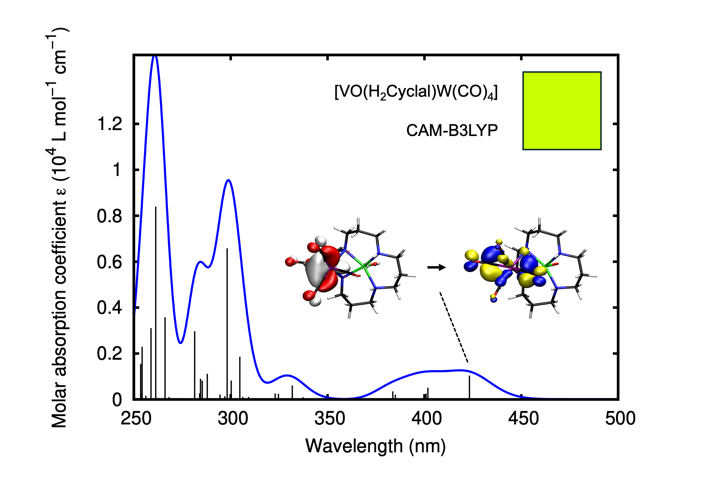


e) f)

**
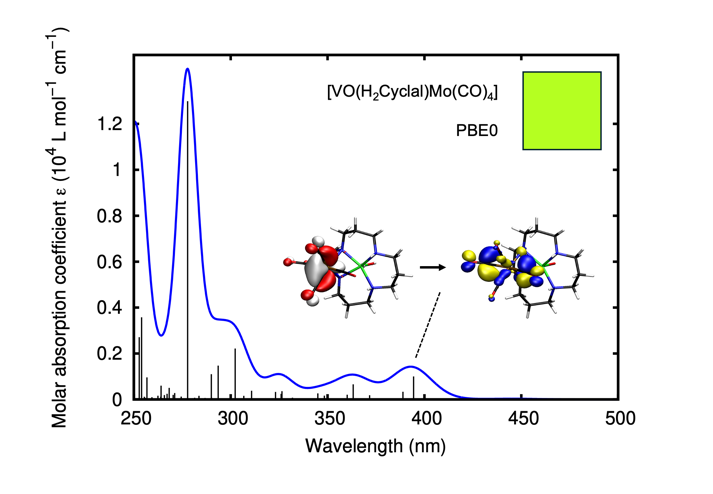

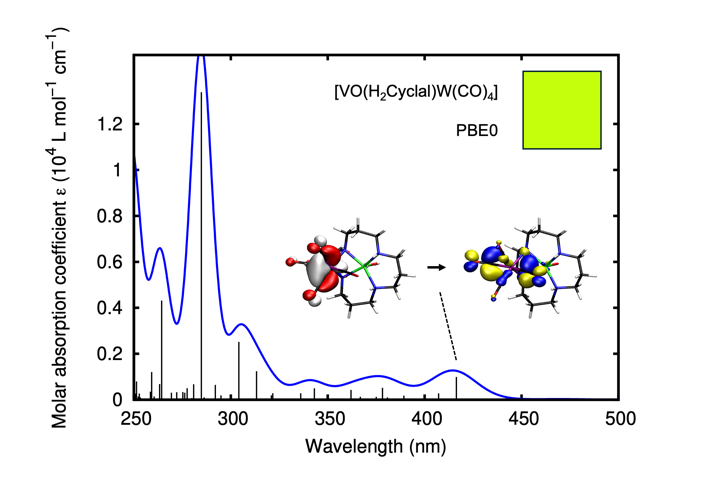
**

**Figure S22.** Simulated UV-Vis spectra of [VO(H_2_Cyclal)Mo(CO)_4_] (left) and [VO(H_2_Cyclal)W(CO)_4_] (right) as obtained at the TD-DFT level using the functional B3LYP (a,b), CAM-B3LYP (c,d), and PBE0 (e,f). Gaussian broadening was employed with a full width at half maximum (FWHM) of 1500 cm^–1^. Also shown are the hole (red/white) and particle (blue/yellow) natural transition orbitals (NTOs)^[S20]^ of the 4d (5d) to carbonyl π* excitation at about 400 nm. The NTOs are displayed for an isovalue of ±0.05 a_0_^–3/2^.

**7. References**

[S1] http://www.jems-saas.ch/

[S2] X-RED32, Data Reduction Program (Version 1.01). Stoe, Darmstadt 2001.

[S3] G. M. Sheldrick, *Acta Crystallogr. A* **2015**, *71*, 3-8.

[S4] DIAMOND, *Crystal and Molecular Structure Visualization*, Crystal Impact GbR, Bonn 2016.

[S5] L. van Gerven, J. Talpe, A. van Itterbeek, *Physica* **1967**, *33*, 207-211.

[S6] J. Nishizawa, H. Shimawaki, Y. Sakuma, *J. Electrochem. Soc.* **1988**, *135*, 1813-1816.

[S7] S. S. Braga, M. P. M. Marques, J. B. Sousa, M. Pillinger, J. J. C. Teixeira-Dias, I. S. Gonçalves, *J. Organomet. Chem.* **2005**, *690*, 2905-2912.

[S8] a) S. Wolf, K. Reiter, F. Weigend, W. Klopper, C. Feldmann, *Inorg. Chem.* **2015**, *54*, 3989-3994. b) M. Liebertseder, S. Wolf, C. Feldmann, *Z. Anorg. Allg. Chem.* **2021**, *647*, 2147-2156.

[S9] TURBOMOLE V7.7.1 2023, a development of University of Karlsruhe and Forschungszentrum Karlsruhe GmbH, 1989-2007, TURBOMOLE GmbH, since 2007; available from <https://www.turbomole.org>.

[S10] C. Lee, W. Yang, R. G. Parr, *Phys. Rev. B* **1988**, *37*, 785-789.

[S11] T. Yanai, D. P. Tew, N. C. Handy, *Chem. Phys. Lett.* **2004**, *396*, 51-57.

[S12] J. P. Perdew, Y. Wang, *Phys. Rev. B* **1992**, *45*, 13244-13249.

[S13] J. Tao, J.P. Perdew, V. N. Staroverov, G. E Scuseria, *Phys. Rev. Lett.* **2003**, *91*, 146401.

[S14] A. Schäfer, H. Horn, R. Ahlrichs, *J. Chem. Phys.* **1992**, *97*, 2571-2577.

[S15] A. Schäfer, C. Huber, R. Ahlrichs, *J. Chem. Phys.* **1994**, *100*, 5829-5832.

[S16] F. Weigend, A. Baldes, *J. Chem. Phys.* **2017**, *133*, 3696.

[S17] A. E. Reed, R. B. Weinstock, F. Weinhold, *J. Chem. Phys.* **1985**, *83*, 735-746.

[S18] J. Pipek, P. G. Mezey, *J. Chem. Phys.* **1989**, *90*, 4916-4926.

[S19] W. Humphrey, A. Dalke, K. Schulten, *J. Molec. Graphics* **1996**, *14*, 33-38.

[S20] R. L. Martin, *J. Chem. Phys.* **2003**, *118*, 4775-4777.
